# Transcriptome analysis of cervical cancer exosomes and detection of HPVE6*I transcripts in exosomal RNA

**DOI:** 10.1186/s12885-022-09262-4

**Published:** 2022-02-11

**Authors:** Anjali Bhat, Joni Yadav, Kulbhushan Thakur, Nikita Aggarwal, Arun Chhokar, Tanya Tripathi, Tejveer Singh, Mohit Jadli, Veeramohan Veerapandian, Alok Chandra Bharti

**Affiliations:** 1 Molecular Oncology Laboratory, Department of Zoology, University of Delhi (North Campus), Delhi, 110007 India; 2 Department of Physiology and Pharmacology, Karolinska Institute, Stockholm, Sweden

**Keywords:** Cervical cancer, Exosome, Human papillomavirus, Next generation sequencing, Transcript profiling, Reverse transcriptase polymerase chain reaction, Oncoprotein E6

## Abstract

**Background:**

Exosomes play a key role in cell-to-cell communication and are integral component of the tumor microenvironment. Recent observations suggest transfer of RNA through tumor-derived exosomes that can potentially translate into regulatory proteins in the recipient cells. Role of cervical cancer-derived exosomes and their transcript cargo is poorly understood.

**Materials and methods:**

The total RNA of exosomes from HPV-positive (SiHa and HeLa) and HPV-negative (C33a) cervical cancer cell lines were extracted and the transcripts were estimated using Illumina HiSeq X. Further, validation of HPV transcripts were performed using RT-PCR.

**Results:**

3099 transcripts were found to be differentially-exported in HPV-positive vs. HPV-negative exosomes (*p* value <0.05). Analysis of top 10 GO terms and KEGG pathways showed enrichment of transcripts belonging to axon guidance and tumor innervation in HPV-positive exosomes. Among top 20 overexpressed transcripts, EVC2, LUZP1 and ANKS1B were the most notable due to their involvement in Hh signaling, cellular migration and invasion, respectively. Further, low levels of HPV-specific reads were detected. RT-PCR validation revealed presence of E6*I splice variant of HPV18 in exosomal RNA of HeLa cells. The E6*I transcripts were consistently retained in exosomes obtained from HeLa cells undergoing 5-FU and cisplatin-induced oxidative stress.

**Conclusion:**

Our data suggests the enrichment of poly-A RNA transcripts in the exosomal cargo of cervical cancer cells, which includes pro-tumorigenic cellular RNA and viral transcripts such as HPV E6, which may have clinical utility as potential exosomal biomarkers of cervical cancer.

**Supplementary Information:**

The online version contains supplementary material available at 10.1186/s12885-022-09262-4.

## Background

Cervical cancer is a disease caused by persistent infection of high-risk Human Papillomavirus (HPV). Despite being a preventable cancer with available clinically-proven prevention tools [1], the disease remains a significant health challenge to women with poor socioeconomic status and is often detected in late stages [2]. Exosomes play an important role in intercellular communication [3]. Tumor-derived exosomes have emerged as mediators of cancer initiation, tumor promotion, metastastic spread, enhanced angiogenesis and drug resistance in a variety of cancers by conditioning the stromal cells and the tumor microenvironment [4, 5]. Therefore, for better understanding of tumor progression, improved exosome based diagnostic and prognostic tools can be developed for effective control and management of cervical cancer. Evidence accumulated over last decade has revealed a strong pro-tumorigenic role of exosomes in cervical cancer. However, role of tumor-derived exosomes and its cargo needs detailed exploration.

Cervical cancer exosomes are known to regulate metastasis [6, 7], angiogenesis [8, 9], drug resistance [10, 11] and tumor progression [12]. Though all three major classes of macromolecules namely DNA, RNA and proteins have been reported in tumor exosomes, the expression of the constituent molecules may greatly differ in comparison to their cell of origin suggestive of selective packaging [13]. Presence of nucleic acids in exosomal cargo is intriguing due to its longer and stronger impact on target cells and is confirmed to be intra-luminal cargo but not an exosome isolation artefact [14]. Multiple RNA types are packaged into the exosomal RNA pool, which include selected portion of the source cell’s RNA spectrum [15]. Small non-coding RNAs are the dominant molecules of the exosomal RNA cargo [7–10, 12]. Similarly, a differential exosomal export of lncRNAs HOTAIR, MALAT1, and MEG3 levels in cervico-lavage samples correlated with cervical cancer progression [16]. Cervical cancer exosomes carried competing endogenous RNA HNF1A-AS1 of miR-34b and promoted cisplatin resistance [11]. Collectively, these molecules have profound effect on the functions of cells that take up the exosomes.

Apart from the regulatory and ribosomal RNA, accumulating evidence highlight the presence of mRNA in exosomal cargo of normal [17, 18], transformed [19] and some cancer cells [14, 20–22]. Although the constitution and biological relevance of this RNA class is poorly defined, the transcripts are hypothesized to participate in horizontal transfer of genetic information as the exosomal mRNA could serve as ready to use templates for *de novo* protein synthesis in the recipient cells [23]. In view of miRNAs’ low quantities and lack of target specificity, functional mRNA may play an equal or perhaps an even more significant role in cell-cell communication by complementing biological signaling pathways, and thus contributing to the disease progression. However, a comprehensive analysis of exosomal mRNA cargo in the cervical cancer cells is currently lacking.

HPV drives cervical carcinogenesis through expression of its viral oncogene E6 and E7 [24] that are coded by a short segment of DNA <1000bp. These genes have an independent transforming potential in cervical epithelial cells [25]. HPV E6/E7 oncogenes have been shown to influence both the content and amount of extracellular vesicles released from HPV positive cells [26, 27]. In an experimental system using keratinocytes transduced with HPV16 E6 and E7 genes, exosomal export of E6 and E7 transcripts was noted that got transferred to non-infected keratinocytes [28]. Therefore, the study of HPV transcripts in exosomal compartment in cervical cancer needs further exploration. In the present study, we screened HPV-negative and HPV-positive cervical cancer exosomes for differentially exported cellular and viral transcripts using NGS analysis.

## Materials and methods

### Materials

Human cervical cancer cell lines with known HPV positivity for HPV type 16 – SiHa, HPV type 18 – HeLa; and HPV-negative C33a were originally procured from ATCC. The materials used in the study have been listed along with their source of procurement. The materials used in the study have been listed along with their source of procurement. DMEM (#AL111-18X500ML), MEM (#AT154), antibiotic solutions (#A018), bovine serum albumin fraction V (#RM10409) were procured from HiMedia Laboratories Pvt. Ltd. (Mumbai, India), Pierce™ BCA Protein Assay Kit (#23225), exosome-depleted serum One Shot™ format (#A2720803), Precision Plus Protein Dual Color Standards from Bio-Rad, USA (#161-0374), High-Capacity cDNA Reverse Transcription Kit (Applied Biosystems™; #4368814), TRIzol RNA Isolation Reagent (#AM9738) were procured from Thermo Fischer Scientific (Waltham, USA). ExoEnrich™ (#PEC-50), ExoLyseP™ (#PEL-25P) were purchased from ExoCan Healthcare Technologies Ltd. (Pune, India). ECL-substrate (#SC-2048) from Santa Cruz Biotechnology, Inc. (Dallas, USA). All the antibodies were procured from Santacruz Biotechnology, Inc. (USA) and Sigma (St. Louis, USA) (Supplementary Table 1) and oligos used in the study were procured from Eurofins scientific (Supplementary Table 2). Millipore PVDF membrane (#HVLP04700), RNAse A (#P4170), and all other reagents unless specified were procured from Sigma.

### Preparation of cell culture conditioned media for exosome studies

Briefly, cells were seeded at 25% confluency (9 × 10^5^) in a 100 mm culture plate containing 10% exosome depleted-FBS and allowed to grow for 4 days. After 4 days, cell culture conditioned medium containing exosomes was harvested and centrifuged at 5000 rpm for 30 min to pellet down remaining cellular debris. The resulting supernatant was then subjected to 0.2 μm filtration step using 0.22 μm membrane (Millipore, MA, USA).

### Isolation of exosomes from cervical cell culture conditioned medium

Exosomes were isolated using commercially available kit, ExoEnrich™ as described previously without any deviation Exosomes were isolated using ExoEnrich™ (PEC-50; ExoCan Healthcare Technologies Ltd, Pune, India) as per manufacturer’s instructions [29]. Briefly, 4 ml of culture conditioned medium was mixed with 100 μl of solution A followed by addition of 2 ml of solution B. The resulting suspension was mixed by gentle pipetting and centrifuged at 3000 rpm for 20 min. The exosome pellet was washed with 1× PBS twice at 3000 rpm, 3 times each. The exosomes were used in downstream analysis or were stored at -20°C until further use.

### Transmission electron microscopy (TEM) of cervical cancer exosomes

TEM analysis of exosome samples was performed according to previously published reports with minor modifications [30, 31]. Freshly isolated cervical cancer exosomes were resuspended in 30 μl of 1X PBS containing 2% paraformaldehyde. Exosomes were prepared for TEM inspection by adsorbing onto Formvar carbon-coated nickel grid for a time period of 1 h. The grids were fixed by 2.5% glutaraldehyde in 0.1 M sodium cacodylate, pH 7.6 for 10 min. After rinsing with sterile distilled water, the grids were contrasted using uranyl-oxalate solution at pH 7 for 5 min, air-dried for 5 min and examined with a JEOL 2100F transmission electron microscope (JEOL Ltd., Tokyo, Japan) operated at 100 kV.

### Isolation of exosomal proteins and immunoblotting for exosome-specific markers

Total exosome proteins were isolated using ExoLyseP™ and immunoblotted as described earlier [29]. Antibodies and their specific dilution in the blocking solution used in the study are described in Supplementary Table 1. Immuno-active bands were detected on an Amersham Imager 600 (GE Life Sciences ABI, Sweden) after 5 min treatment of the blot with enhanced chemiluminescence detection kit.

### Cellular and exosomal RNA isolation and quantification

Exosome pellets were removed of exterior DNA by DNaseI digestion and treated with RNase to remove outer RNA as using TRIzol reagent as per manufacturer’s instructions. Trizol was added to 10^6^ cells and to exosomes isolated from 4 ml of conditioned medium normalized per 10^6^ cells from all the cell lines followed by homogenization and treatment of chloroform. The suspension was allowed to stand at RT for five minutes and then subjected to centrifugation at 12,000 rpm for 15 minutes as per manufacturer’s instructions. The aqueous layer was taken off and collected in a new tube. RNA was precipitated using isopropanol at -80°C for overnight and pelleted at 12,000 rpm for 20 min. Isolated RNA was dissolved in a minimum of 20 μl of nuclease free water. RNA was quantified spectrophotometrically using NanoQuant T^m^ (Tecan). For RNA visualization silver staining was performed on non-denaturing PAGE as described earlier [29]. Briefly, RNA was isolated from exosome preparations (from 4 ml culture conditioned medium) and subjected to polyacrylamide gel electrophoresis for 3 h at 100 V in 0.5× Tris-borate buffer. The gel was fixed in 150 ml of 50% Methanol 5% Acetic acid for 20 min followed by washing with 50% Methanol and water for 10 min. The gel was sensitized using 0.02% sodium thiosulphate solution for 1 min followed by a silver reaction using 0.1% Silver Nitrate in 0.08% formalin (37%) for 20 min and developed using 2% sodium carbonate with 0.04% formalin. The RNA concentrations were determined by Qubit (Thermo Fisher Scientific, USA) and the RIN for sequencing analysis.

### Transcript analysis by reverse transcriptase (RT)-PCR

A minimum of 2 μg of sample RNA was used for cDNA synthesis in a 20 μl reaction using High-Capacity cDNA Synthesis Kit as described previously [32]. PCR was performed for amplification of specific genes on Veriti Thermal Cycler Pro from Applied Biosystems in a 10 μl reaction volume. Primer sequence with annealing temperature is described in Supplementary Table ST2.

### Modulation of exosomal HPV RNA cargo by cytotoxic drugs

HPV-positive cervical cancer cells were exposed to cytotoxic drugs, 5-FU and cisplatin. For this, 2.5× 10^3^ cells were seeded in a 96-well plate and grown overnight to allow attachment. Subsequently, cells were treated with IC_50_ doses of 5-FU (17.58 μM: SiHa; 15.31 μM: HeLa) and cisplatin (24.14 μM: SiHa; 6.82 μM: HeLa) for 48 h and the exosomes were isolated from the conditioned media of treated cells. The exosomal RNA was isolated and examined for modulation of HPV transcripts by type-specific HPVE6 and E7 RT-PCR.

### RNA isolation, NGS library preparation and sequencing

RNA isolation, NGS library preparation and high-throughput sequencing was outsourced to Clevergene Biocorp. Pvt Ltd. (Bengaluru, Karnataka, India). Total RNA in the exosomes was isolated using the TRIzol reagent according to the manufacturer’s instructions. The RNA concentrations were determined by Qubit (Thermo Fisher Scientific, USA) and the RIN (RNA Integrity Number) was checked by Agilent 2100 Bioanalyzer (Agilent Technologies). For each library preparation, 1 ng of total RNA from each sample was used. NEBNext Ultra II RNA Library Prep Kit for Illumina (# E7775) was used. Total cDNA was ligated with P7 and P5 adapter sets. The PCR products were gel purified and their quality confirmed by Bioanalyzer (Supplementary Fig. SF1). Paired-end sequencing was performed on these libraries using an Illumina HiSeq X platform with a minimum of 25-30 million reads per sample.

### Sequence data QC

NGS data quality was checked using FastQC (http://www.bioinformatics.babraham.ac.uk/projects/fastqc/) and MultiQC [33]. The data was checked for base call quality distribution, % bases above Q20, Q30, %GC, and sequencing adapter contamination (Supplementary Table ST3). Raw sequence reads were processed to remove adapter sequences and low-quality bases using fastp [34].

### Alignment and expression analysis

The QC passed reads were mapped onto indexed Human (GRCh38.p7), HPV16 and HPV18 reference genome using STAR v2 aligner [35]. Uniquely mapped reads were used for transcripts assembly. For assembling transcripts, StringTie was used with default parameters [36]. Assembled transcripts of all the samples were merged into a single gtf file using the String Tie merge option. The merged gtf was compared and annotated with reference gff using gff compare [37]. The relationship between the assembled transcripts and closely related reference transcripts along with the key for these codes is provided in Supplementary Fig. SF2.

### Differential transcript abundance analysis

Differential analysis was carried out using the DESeq2 [38]. The read counts were normalized (variance stabilized normalized counts) and differential enrichment analysis was performed. For HPV-negative vs. HPV-positive analysis, C33a was used as reference and SiHa and HeLa exosomal transcripts were used as test groups. For HPV16 vs. HPV18 analysis, SiHa was used as reference and HeLa exosomal transcripts were used as test group. Transcripts with absolute log2 fold change ≥ 1.5 and p-value ≤ 0.05 were considered significant. The profile of differentially exported transcripts across the samples was evaluated using volcano plots. The transcripts that showed significant differential expression for human reference genome (GRCh38.p7) were used for Gene Ontology (GO) and KEGG pathway enrichment analysis.

### GO and pathway analysis

Enrichment analysis for Biological Process (BP), Molecular Function (MF), Cellular Component (CC) and KEGG Pathways was performed using Cluster Profiler R Bioconductor package [39, 40]. GO and pathway terms with adjusted *p*-value ≤ 0.05 were considered significant. To visualize the GO enrichment results, GO plot, R package was used [41]. GO plot package calculated z-score using the following formula:

$$\mathrm{z}\ \mathrm{score}=\left(\mathrm{up}-\mathrm{down}\right)/\sqrt{count}$$

where up is the number of up-regulated genes in a GO term and similarly down represents number of down-regulated genes in the GO term. The z-score provides a rough idea about the expression profile of genes within a GO term. The pathways were visualized using the Pathview package to check the differential expression level of the genes in the pathway [42].

## Results

### Characterization of cervical cancer exosomes

**Fig. 1:**
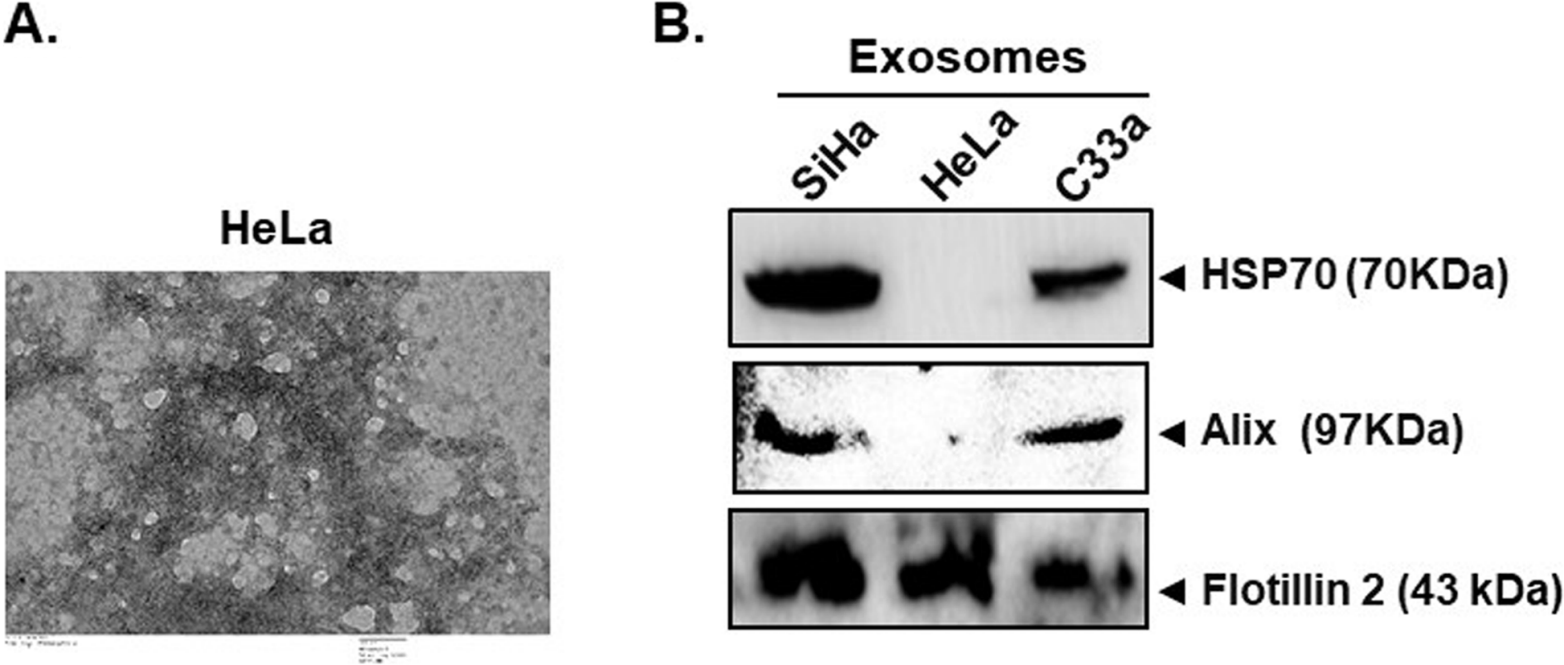
Characterization of exosomes derived from cervical cancer cell lines. **A** Morphometric measurement of cervical cancer exosomes using transmission electron microscopy. Exosomes from indicated cervical cancer cell lines were isolated from culture conditioned medium and prepared for evaluation as described in methods. **B** Immunoblotting for exosome-specific markers. Exosomal lysates (50 μg/lane) were resolved on SDS-PAGE, electrotransferred and immunoblotted for Flotillin 2, HSP70, and Alix

In order to profile the mRNA content of exosomes, the purified exosomes were first visually confirmed by TEM, which revealed the presence of typical cup shaped bilayer vesicles homogenous in size and morphology (Fig. 1A). Further, immunoblotting for exosomal lysates showed the presence of exosome-specific markers like Flotillin 2, HSP70, and Alix (Fig. 1B). The presence of these markers is consistent with the previously reported characteristics of exosomal particles.

### Assessment of exosomal RNA quality and integrity

**Fig. 2:**
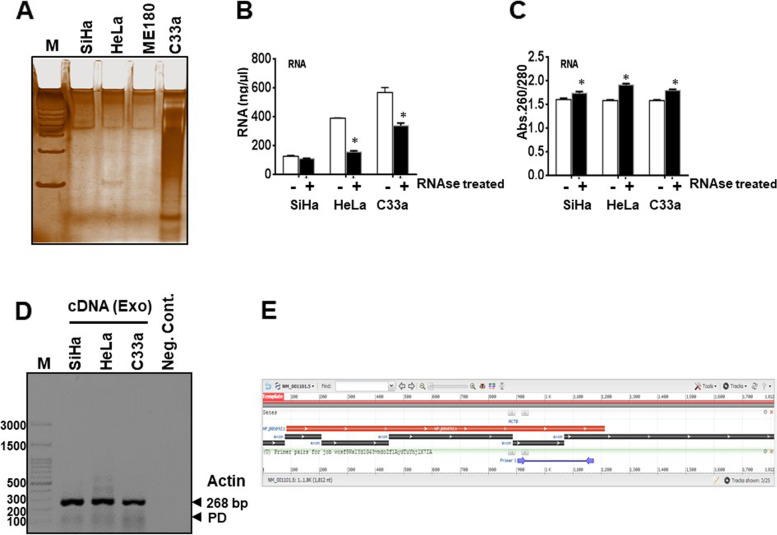
Qualitative and quantitative assessment of isolated exosomal RNA. RNA estimation in cervical cancer exosomal cargo. **A** Representative silver staining image of RNA isolated from cervical cancer exosomes. **B** Spectrophotometric quantification of isolated RNA. Exosomes were pre-treated with RNase before isolation of intra-luminal exosomal RNA to eliminate non-exosomal RNA contamination. **C** Quality estimation of exosomal RNA using Abs.260/280 ratio. **D** PCR amplification of β-actin in cDNA derived from exosomal RNA. Exosomal RNA (2 μg/10 μl reaction) from indicated cervical cancer cell lines was used for RT-reaction followed by β-actin PCR. **E** NCBI Primer-BLAST graphical view of human β-actin mRNA and location of the primers used for RT-PCR on β-actin transcript

Qualitative and quantitative estimation of the isolated exosomal RNA was performed using silver staining (Fig. 2A) and spectrophotometry (Fig. 2B and C). The exosomal RNA yield ranged from 100 ng/μl to 600 ng/μl from exosomes without any pre-treatment. However, there was a significant decline upto 60% in RNA yield when exosomes were pre-treated with RNase (Fig. 2B). In contrast, the RNase – treatment resulted in an improved A260/A280 ratio (Fig. 2C). PCR amplification of β-actin gene carried out using exosomal RNA from both HPV-positive and HPV-negative cervical cancer cell lines indicated an amplifiable RNA cargo in all the exosomal RNA preparations (Fig. 2D). The location of the primers/amplicon as deduced by NCBI Primer-BLAST was 904-1171 and the full transcript length of β-actin was 1812 nt (Fig. 2E). Therefore, all the exosomal RNA carried at least 900 nt long transcripts with an intact 3’ end poly-A tail. Analysis of exosomal transcript length with intactness/integrity across different tumor types, has been summarised in Table 1.

**Table 1:** Estimation of intactness or integrity of transcript length using reverse transcriptase mediated cDNA preparation using 3’ end followed by region specific PCR

Tumor type (Reference)	Transcript examined in exosomal RNA	Full length mRNA (bp)	Nucleotide position showing amplification	Product length (bp)	Transcript coverage (nt)
**Glioblastoma** [14]	GAPDH	1231	82-256	175	1150
	EGFR/EGFRvIII PCR1	1575	216-1447	1232	1360
	EGFR/EGFRvIII PCR2	1775	262-1414	1153	1514
**Colorectal cancer** [20]	β-actin	1812	353-479	127	1460
	β-catenin (CTNNB1)	3488 3197	206-471 206-339	266 134	3283 2992
	RAB13	1164	182-254	73	983
	CXCR4	1904	116-188	73	1789
	MYC	3721	371-440	70	3351
**Breast Cancer** [21]	EEF1A1	3512	1329-1438	110	2184
	FTH1	1203	440-573	134	764
	FTL	871	384-529	146	488
	RAB13	1347	785-891	107	563
	RPL28	4209	215-338	124	3995
**Prostate cancer** [49]	GAPDH	1285	950-1050	101	336
**Cervical cancer (present study)**	β-actin	1812	904-1171	268	908

### Abundance analysis of exosomal transcripts

**Fig. 3:**
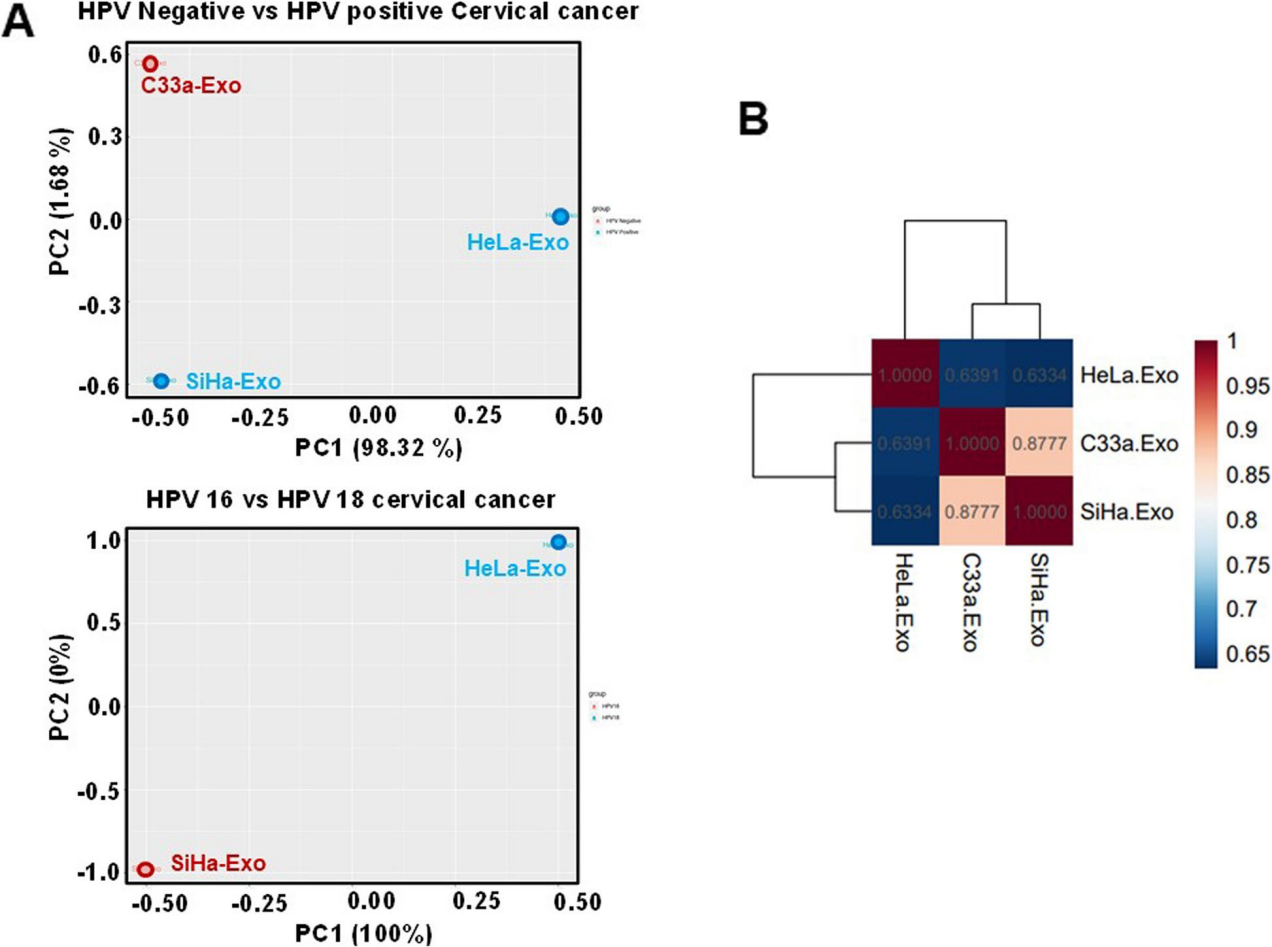
Analysis of overall distribution of transcript content in exosomal compartment of HPV-negative and HPV-positive cervical cancer cells. **A** Principal Component Analysis (PCA) of exosomal transcripts in HPV-positive vs. HPV-negative cervical cancer cells (upper panel) and HPV16-positive SiHa cells vs. HPV18-positive HeLa cells (lower panel) **B**. Pearson’s Correlation plot

Further, mRNA sequencing was performed on total RNA extracted from exosomes to analyse the exosomal transcript content and abundance. RNA integrity assessed on Agilent 2100 Bioanalyzer, indicated the presence of 100-1000 bp fragments in cDNA prepared (Supplementary Fig. SF1). The QC passed reads mapped onto the indexed combined reference genome of Human (GRCh38.p7), HPV16 and HPV18 on average showed 96.88% of the reads aligned onto the combined reference genome, whereas 1.07 and 1.60% of the reads aligned onto HPV16 and HPV18 reference genomes (Supplementary Table ST4). Out of total 606,022 transcripts evaluated, expressed transcripts in each sample with at least 1 mapped read ranged from 533,123 in C33a (87.9%) to 514,553 in SiHa (84.9%) and 245,988 in HeLa (40.59%) exosomes. The expression similarity between samples as determined by Principal Components Analysis showed that all three samples clustered into three separate groups, with no significant outliers (Fig. 3A). Further, the Pearson’s correlation values indicated that HPV16-positive SiHa and HPV-negative C33a exosome samples were positively corelated with each other than with HPV18-positive in both the comparison sets (Fig. 3B).

### Differential transcript analysis

**Fig. 4:**
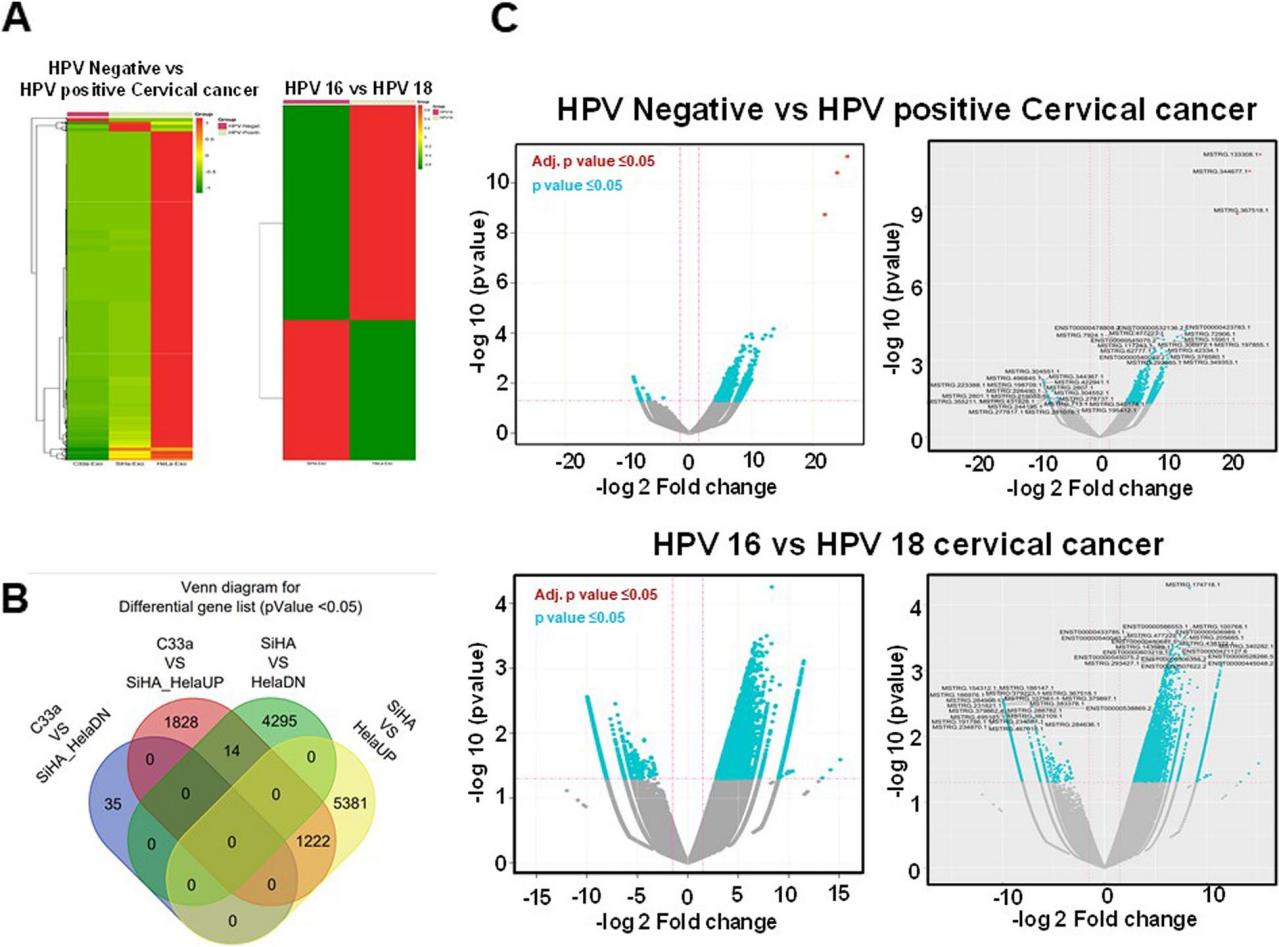
Differentially expressed transcript profile of cervical cancer exosomes. **A** Distinct mRNA expression profiles in HPV-positive vs. HPV-negative and HPV16-positive vs HPV18-positive. The inclusion criteria of these transcripts was a 2-fold difference of log2 (fold-change) in either direction with a *p* value <0.05. Red signal, represents higher relative expression as compared to green signal**. B** Quantitative Venn diagram showing differentially expressed gene. **C** Volcano plot showing overall clustering of differentially expressed exosomal transcripts in HPV-positive vs. HPV-negative and HPV16-positive vs. HPV18-positive (left panels). Volcano plot showing distinct clustering of top 20 transcripts (right panels). Blue color indicates log2 fold change ≥1.5 and *p* value ≤0.05. Red dots indicate absolute log2 fold change ≥1.5 and FDR/adjusted *p* value ≤0.05. Gray lines indicate the marginal lines separating DEGs from non-DEGs, with the horizontal lines denoting the p value threshold (*p* ≤ 0.05) and vertical lines the fold change cut off (≥ 1.5 or < 0.67)**.**

Hierarchical clustering showed profiles of exosomal transcripts and indicated that SiHa and C33a exosomal transcriptome was similarly clustered as compared to SiHa and HeLa (Fig. 4A). Of 3,099 differentially-exported transcripts, 3,064 were significantly upregulated and 35 were downregulated in HPV-positive exosomes. Next, in the comparison set of exosomes derived from HPV18-positive cells, 10,912 transcripts were differentially exported. Among these, 6,603 transcripts were significantly upregulated and 4,309 were downregulated. Fourteen transcripts were found exclusively upregulated in SiHa exosomes, and 1,222 transcripts in HeLa exosomes (Fig. 4B). The expression profile of differentially-exported exosomal transcripts across HPV-negative vs. HPV-positive and HPV16- vs. HPV18-positive groups are represented in volcano plots (Fig. 4C). Overall differential expression of transcripts with top 20 hits, between the indicated comparison sets were identified (Fig. 4C right panels) and listed in Table 2.

**Table 2:** List of top 20 upregulated and downregulated transcripts in exosomal RNA from HPV-positive cells

Fragment Id	Gene Name	Gene Description	Enrichment Score (FPKM)			***P*** value
			C33a-Exo	SiHa-Exo	HeLa-Exo	
**Upregulated**						
MSTRG.367518.1	TBC1D9	TBC1 domain family member 9	0.0	318.0	0.0	1.87E-09
MSTRG.344677.1	EVC2	EvC ciliary complex subunit 2	0.0	1313.3	0.0	3.99E-11
MSTRG.133308.1	-	-	0.0	0.0	4341.5	8.89E-12
ENST00000423783.1	ACTG1P24	-	0.0	3.8	3570.2	6.77E-05
ENST00000478808.2	USP30-AS1	-	3.2	10.6	7285.6	0.000154
ENST00000532136.2	OR5AM1P	-	4.7	30.0	9612.9	0.000111
MSTRG.477223.1	RP11-11N9.4	-	17.4	195.9	20881.6	0.000136
MSTRG.72906.1	-	-	0.0	28.8	1423.9	0.00012
MSTRG.15951.1	NFIA	nuclear factor I A	0.0	1.9	2700.7	0.00013
MSTRG.306972.1	-	-	3.2	5.0	5714.5	0.000327
MSTRG.197855.1	-	-	0.0	40.1	1029.7	0.000169
MSTRG.42334.1	-	-	0.0	294.2	154.7	0.000437
MSTRG.376580.1	-	-	0.0	16.3	633.7	0.000542
MSTRG.349353.1	-	-	0.0	50.7	414.9	0.000613
MSTRG.292085.1	CH507-513H4.1	-	1.6	2.5	2853.5	0.000539
ENST00000540040.2	MTRNR2L1	MT-RNR2-like 1	58.5	380.0	35309.4	0.000563
MSTRG.62777.1	-	-	4.7	47.6	3485.3	0.000487
MSTRG.117243.1	ANKS1B	ankyrin repeat and sterile alpha motif domain containing 1B	3.2	27.5	2961.0	0.000436
MSTRG.7924.1	LUZP1	leucine zipper protein 1	1.6	83.9	1241.0	0.00037
ENST00000545075.2	MTRNR2L10	MT-RNR2-like 10	24.5	213.5	19438.8	0.000315
**Downregulated**						
MSTRG.496845.1	RP11-661A12.12	-	69.6	0.0	0.0	0.016636
MSTRG.304551.1	APOBEC3A	apolipoprotein B mRNA editing enzyme catalytic subunit 3A	104.3	0.0	0.0	0.005624
MSTRG.223388.1	-	-	59.3	0.0	0.0	0.024676
MSTRG.198709.1	-	-	61.6	0.0	0.0	0.022445
MSTRG.226490.1	CTD-2189E23.1	-	60.9	0.0	0.0	0.023159
MSTRG.344367.1	-	-	95.6	0.0	0.0	0.007161
MSTRG.2801.1	-	-	58.5	0.0	0.0	0.025481
MSTRG.219083.1	OACYLP	-	58.5	0.0	0.0	0.025481
MSTRG.355211.1	UGT2B17	UDP glucuronosyltransferase family 2 member B17	57.7	0.0	0.0	0.02632
MSTRG.431828.1	-	-	56.9	0.0	0.0	0.027195
MSTRG.244195.1	-	-	55.3	0.0	0.0	0.029056
MSTRG.277817.1	AGAP1	ArfGAP with GTPase domain, ankyrin repeat and PH domain 1	56.9	0.0	0.0	0.027195
MSTRG.281076.1	SNAP25-AS1	SNAP25 antisense RNA 1	50.6	0.0	0.0	0.035696
MSTRG.195412.1	MYO15A	myosin XVA	55.3	0.0	0.0	0.029056
MSTRG.545174.1	-	-	56.9	0.0	0.0	0.027195
MSTRG.278737.1	THAP4	THAP domain containing 4	83.8	1.3	0.0	0.028536
MSTRG.304552.1	APOBEC3A	apolipoprotein B mRNA editing enzyme catalytic subunit 3A	88.5	0.6	0.0	0.015573
MSTRG.2807.1	-	-	86.9	0.0	0.0	0.009282
MSTRG.422941.1	FILIP1	filamin A interacting protein 1	93.3	0.0	0.0	0.007671

### Functional analysis of the exosomal transcripts

**Fig. 5:**
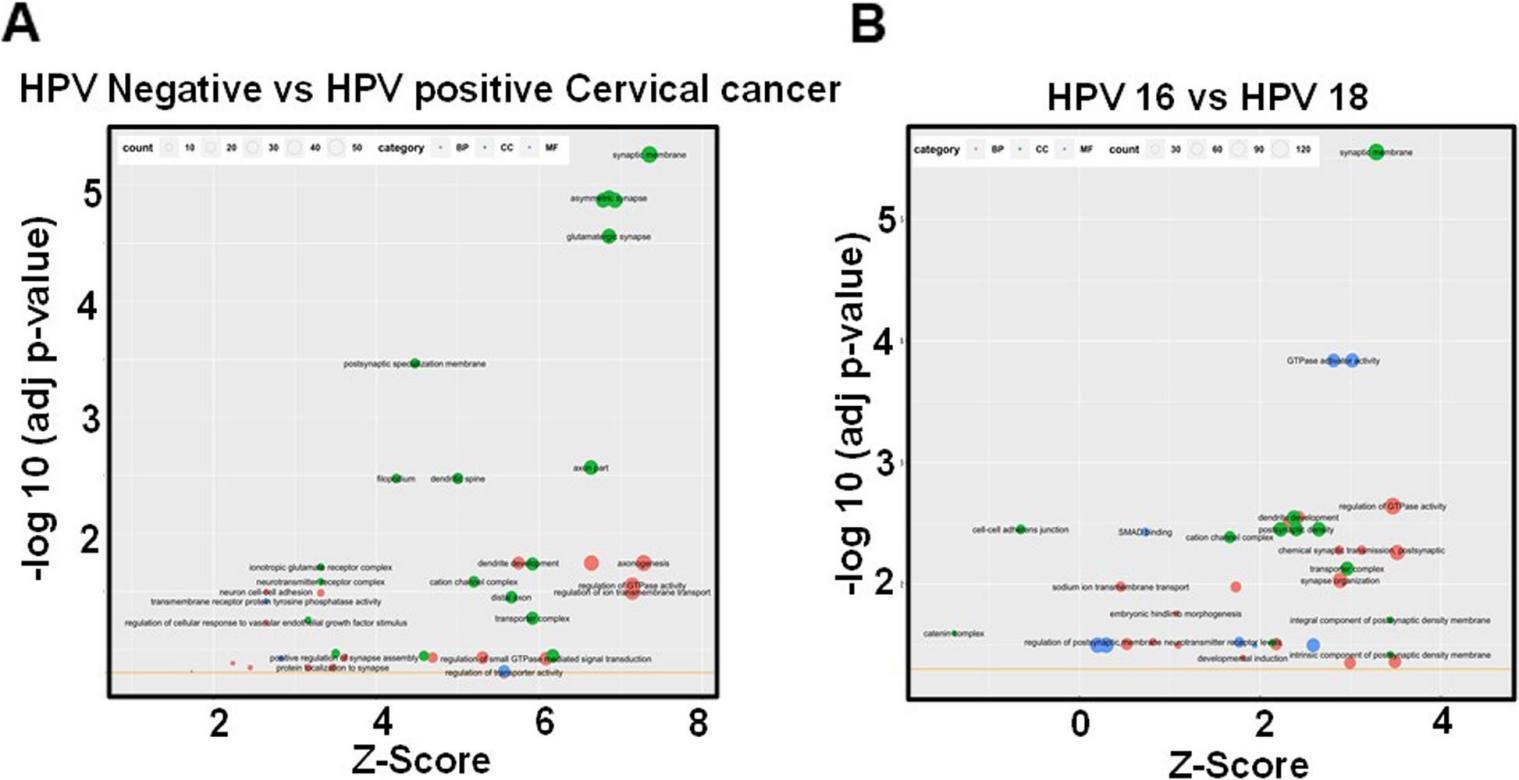
Functional clustering of the differentially expressed transcriptomic profile of exosomes of cervical cancer cell lines as per GO terms. Gene Ontology (GO) clustering of differentially-expressed exosomal transcripts in HPV-positive vs. HPV-negative (**A**) and HPV16-positive vs. HPV18-positive exosomes (**B**) using Bubble plot of the enriched GO terms. GO terms both with a *p* value <0.05 were selected. The size of the bubble is proportionate to the number of genes involved in the GO term. Bubble color code: red – Biological Process (BP); blue – Molecular Function (MF); green – Cellular Component (CC)

**Fig. 6:**
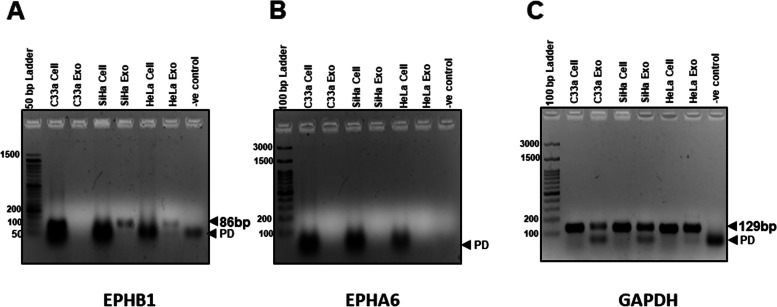
Detection of EPHB1 and EPHA6 transcripts in cervical cancer exosomes. Representative agarose gel photographs of specific PCR amplicons of EPHB1 and EPHA6 in SiHa, HeLa and C33a cells along with their respective exosomal RNA. RT-PCR reaction performed on cDNA prepared from (2 μg/10 μl reaction) of genomic or exosomal RNA. Exo.- exosomal cDNA, M-marker, PD-primer dimer

Gene Ontology (GO) enrichment analysis revealed a total of 54 enriched GO categories using a *p* value cut-off ≤ 0.05 for 3,099 differentially-exported transcripts in HPV-positive exosomes (Fig. 5A). The significantly altered transcripts belonged to 23 BP categories, 6 MF categories and 25 CC in exosomal compartment of HPV-positive cells. The differentially exported transcripts showed strong association with regulation of ion transport, GTPase activity and axonogenesis among the major BPs; regulation of transporter and tyrosine phosphatase receptor activity among the major MFs; and asymetric synapse, synaptic membrane and glutamatergic synapse among the major CCs. Whereas, among 10,912 differentially-exported transcripts showed enrichment of 56 GO, out of which the transcripts belonged to 28 BPs, 12 MFs and 16 CCs categories (Fig. 5B). Regulation of GTPase activity, post synaptic transmission, sodium ion transmembrane transport were the major BPs, cation channel complex, cell-cell adherens junctions, post synaptic density, emerged as top CCs; and GTPase activity, regulation of neurotransmitter activity were the major MFs that carried the highest z-score. KEGG pathway analysis of differentially-exported transcripts identified 9 pathways in HPV-positive exosomal transcript sets (Supplementary Fig. SF3A-SF3I). The exported transcripts were mainly associated with the regulation of the calcium signaling, cAMP signaling, axon guidance, leukocyte transendothelial migration, circadian entrainment, long term potentiation, glutamatergic synapse, GnRH secretion and morphine addiction. To further validate the observation, we performed RT-PCR to detect the transcripts encoding EPHB1 and its isoform EPHA6, well known transcripts regulating axonogenesis and neuronal growth in exosomes of cervical cancer cells. Our RT-PCR data confirmed detectable levels of EPHB1 transcripts in exosomes of HPV positive cells, however, the EPHA6 transcripts were not detected in these exosomes (Fig. 6).

### Identification of HPV transcripts in exosomal cargo

**Fig. 7:**
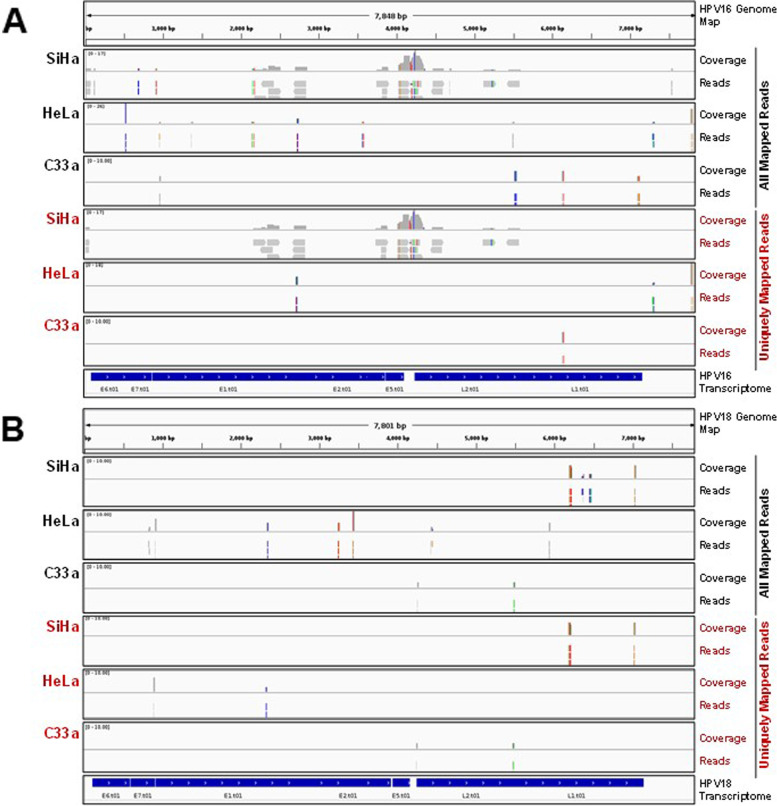
Enrichment of HPV-related reads in exosomal transcripts. Integrated Genome Viewer snapshots showing mapping of all mapped reads and uniquely mapped reads detected in SiHa and HeLa exosomes to HPV16 (**A**) or HPV18 (**B**) reference genomes, respectively

Next we examined, the exosomal compartment for export of HPV transcripts using all mapped and uniquely mapped reads on Integrative Genomics Viewer (IGV). The data showed presence of low abundance reads sparingly scattered over the reference transcriptomes of HPV16 and HPV18 in SiHa and HeLa exosomes, respectively (Fig. 7A, B). However, these number were consistently higher than the C33a exosomal reads which seldom mapped to HPV16 or HPV18 genomes. Among others, E5 region showed highest representation in SiHa exosomes. However, a few reads were also detected in regions corresponding to HPV E6 and E7 regions in both SiHa and HeLa exosomal compartments.

### Evaluation of HPV oncogene transcripts in exosomal cargo

**Fig. 8:**
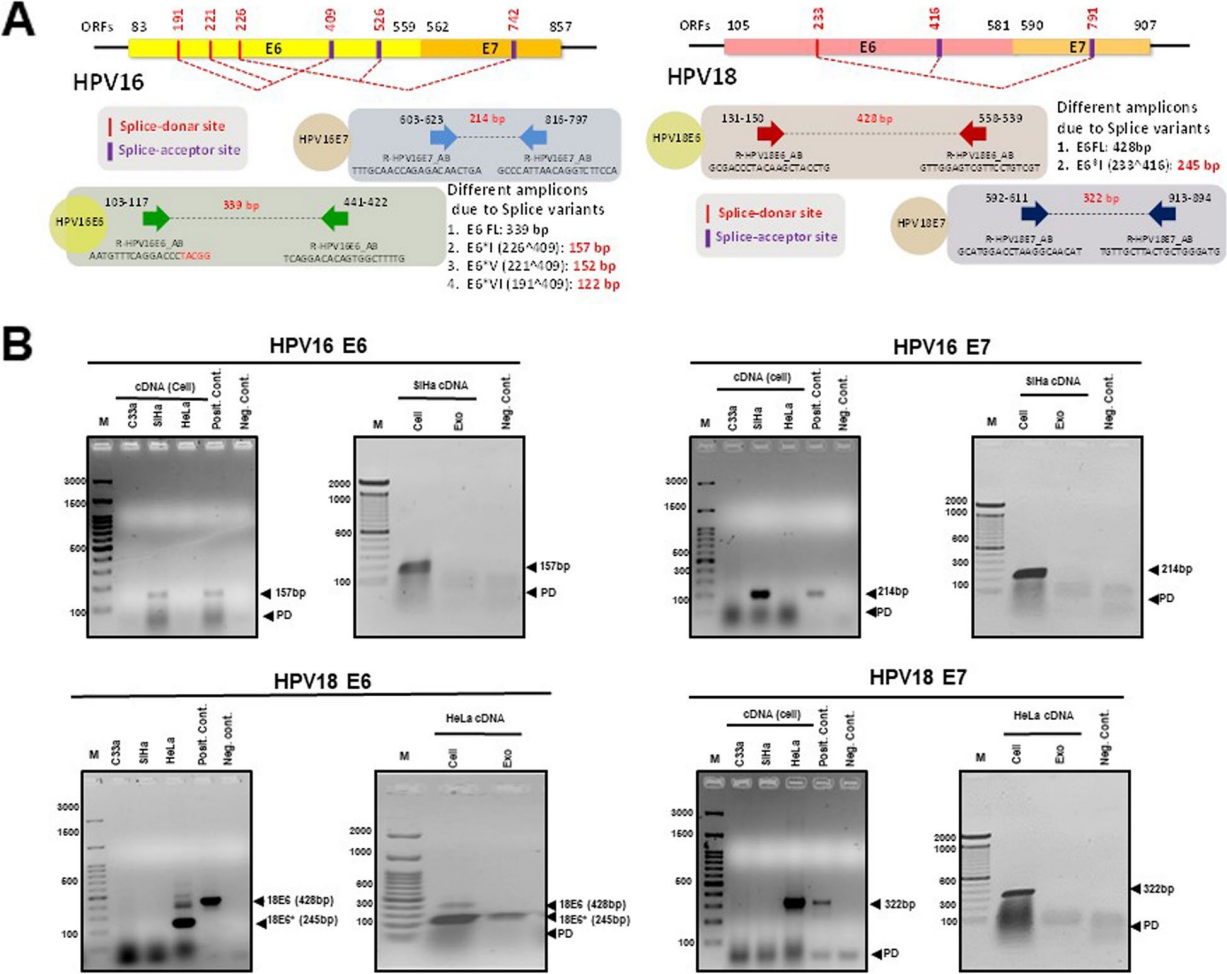
Detection of HPV encoded transcripts in cervical cancer exosomes. **A** Schematic representation of HPV16 and HPV18 genomes and the primer coordinates for targeted amplification of E6 and E7 transcripts. Primers are marked as solid arrows with their nucleotide binding position, their corresponding sequences and amplicon size. The size of amplicons from full length transcripts and spliced variants are indicated in respective panels (in red font). **B** Representative agarose gel photographs of specific PCR amplicons of HPV16/18 E6 and E7 in SiHa and HeLa cells and in their respective exosomal RNA. RT-PCR reaction performed on cDNA prepared from (2 μg/10 μl reaction) of genomic or exosomal RNA. Exo.- exosomal cDNA, M-marker, PD-primer dimer

**Fig. 9:**
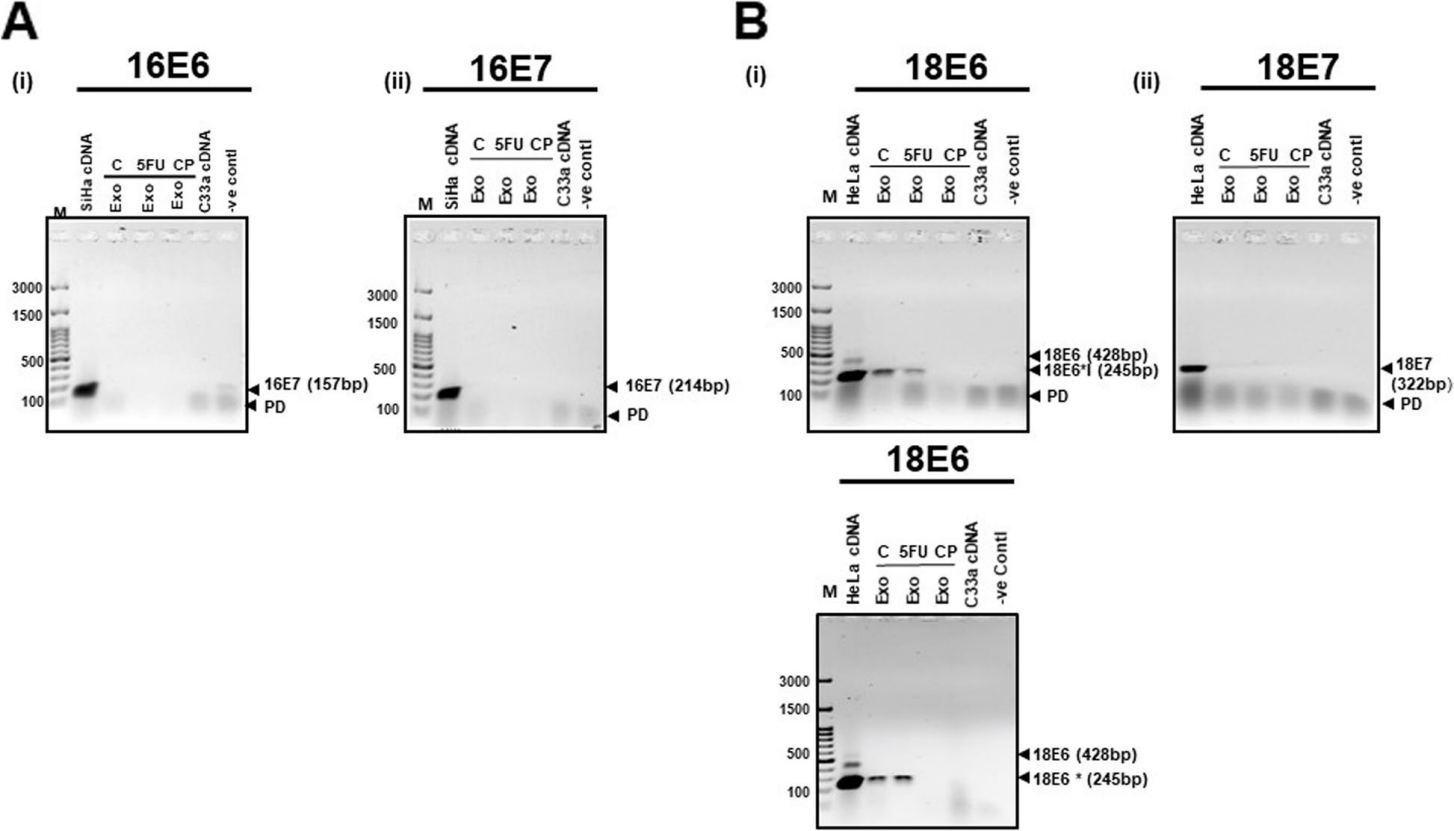
Detection of HPVE6, and E7 transcripts in exosomal RNA of cervical cancer cells undergoing genotoxic stress. Gel images showing E6 and E7-specific amplification in cDNA prepared from exosomal RNA of 5-FU or cisplatin-treated SiHa (**A**) and HeLa cells (**B**). RT-PCR was performed on exosomal RNA (2 μg/reaction). Cellular RNA from corresponding untreated cells was used as a reference. C33a cDNA was used as a negative control. Exo.-exosomal cDNA, M-marker, Neg: template free reaction, PD-primer dimer

In order to validate the presence of HPV E6 and E7 in exosomal transcripts, RT–PCR was performed on the RNA isolated from cervical cancer exosomes. The PCR primers that specifically target either E6 or E7 regions of HPV16 or HPV18 in a type-specific manner could amplify full length transcripts and different splice variants as indicated (Fig. 8AR). Notably, both the cervical cancer cell lines showed type-specific presence of E6 and E7 transcripts in their cellular compartment; however, we could not detect E6 and E7 transcripts in exosomes of SiHa cells, when loaded with the cDNA from cells as internal control (Fig. 8BR). In contrast, HeLa exosomes consistently showed amplification of 245 bp region suggesting presence of HPV transcripts corresponding to E6*I (233^416) that was found overrepresented in HeLa cells. However, full length HPV18 E6 (428 bp) that was expressed in HeLa cellular compartment in low abundance was undetectable in exosomal compartment. Further, to check if the cytotoxic drugs influence the export of HPV transcripts in cervical cancer exosomes, cervical cancer cells were treated with either 5-FU or cisplatin at the IC_50_ dose [SiHa: (17.58 μM; 5-FU and 24.14 μM: cisplatin); HeLa: (15.31 μM; 5-FU and 6.82 μM: cisplatin)] for a period of 48 hrs. Against our anticipation, we did not detect HPV E6 or E7 in SiHa or HeLa exosomes post-drug treatment (Fig. 9A). Contrarily, we observed a decline in the HPVE6*I transcripts in exosomes of drug-treated HeLa cells (Fig. 9B).

## Discussion

The investigation showed presence of pro-tumorigenic transcripts in cervical cancer exosomes. The exosomal RNA contained poly A-tailed transcripts that successfully reverse transcribed and were PCR amplifiable. NGS data showed that upto 88% of human transcriptome with at least one mapped read [533,123 in C33a (87.9%) to 514,553 in SiHa (84.9%) and 245,988 in HeLa (40.59%)] was represented in the exosomal cargo. Analysis of inter-sample variation revealed a small subset of differentially-exported transcripts in HPV-negative and HPV-positive cell-derived exosomes. In contrast, the exosomal cargo of HPV16- and HPV18-positive cells showed a large number of differentially-exported transcripts. Our attempt to find HPV-specific transcripts in exosomal compartment exhibited low abundance reads that mapped to different regions of HPV transcriptome. RT-PCR validation confirmed the presence of HPV E6*I in HeLa exosomes.

Herein, we report for the first time the export of mRNA in the cervical cancer exosomes from tumor cells. Export of PCR-amplifiable tumor-derived mRNA has been previously reported in glioblastoma [14], colorectal [20], breast [21], and prostate cancer [22] as well as in transfomed cell line HEK 293T [19]. However, a quantitative assessment of the RNA yield from exosomes was consistently lacking in all these studies. Our data demonstrated a 4-5 fold difference in exosomal RNA yield among different cell lines tested. Although exosomal release is a regulated process controlled by GPCR’s [43], the reason for a differential content of packaged exosomal RNA is not known and may primarily depend on the metabolic state of the parent cell. Total RNA from untreated exosomes was found to contain cell-free nucleic acids that could have influenced the study outcome. RNase pretreatment reduced the total exosomal RNA yield by 7% as reported by an earlier study [14]. The decrease in RNA yield upto 60% on treatment with RNAse perhaps indicates the presence of RNA from cell free nucleic acids. Nevertheless, RNase pre-treatment resulted in an improvement of A260/A280 ratio. Silver staining of RNA from these exosomes revealed a broad range of RNA sizes. As reported earlier [14], the exosomal RNA lacked characteristic rRNA bands routinely detected in the cellular RNA preparations.

The length of cervical cancer exosomal mRNA varied between 170 and 984 nt. Maximum length of mRNA reported in exosomes is still debatable. High throughput studies failed to discriminate between full length and fragmented mRNA’s in exosomes [19, 21]. The intact cellular mRNAs on an average measure 2000 nt with a range of 350 and 12,000 nt [44]. Earlier, exosomal mRNA appeared predominantly fragmented and 3’-UTRs-enriched [45]. However, a comparative RNA length analysis carried out on glioma stem cell and their respective exosomes showed a specific enrichment of 3’UTR transcripts, without any preference for shorter transcripts in the exosomal fractions [46]. RT-PCR using cDNA intermediates derived from poly-A transcripts can permit a direct estimation of the exosomal mRNA length. These observations suggest presence of intact, biologically-active transcripts like beta actin in the exosomal compartment. The presence of beta actin and GAPDH has already been reported in the mouse and human exosomes respectively (http://www.exocarta.org/).

Other studies using alternate NGS library preparation strategies have shown that 58% of the non-rRNA reads of breast cancer exosomal RNA could map to known genes [21]. Studies based on high resolution microarray platforms [19, 20] also showed presence of several transcripts in exosomes; however, the magnitude of different transcripts reported in exosomal cargo was comparatively lesser.

PCA plot and corelation similarity index of the normalised read counts (C33a vs SiHa: 0.8777> C33a vs HeLa: 0.6391> SiHa vs HeLa: 0.6334) showed distinct qualitative and quantitative transcript profile of all the three exosomal samples. SiHa and C33a exosomal samples displayed lower intra sample variance as compared to SiHa vs. HeLa exosomal transcripts. Such pattern might be an outcome of cancer cell origin. C33a (ATCC-HTB-31) and SiHa cells (ATCC-HTB-35) originated from squamous cell carcinoma, whereas HeLa cells (ATCC-CRM-CCL-2) had adenocarcinoma origin. Both the diseases are known to express a distinct molecular profile and separate clinico-pathological spectrum [47].

Functional gene analysis using GO and pathway analysis using KEGG unexpectedly revealed enriched transcripts playing key roles in several biological and molecular functions related to nerve growth, neuronal functions and cell migration. Differentially exported transcripts showed the gene annotations of regulating synaptic membrane potential, calcium signaling, cAMP signaling, axon guidance, leukocyte transendothelial migration, circadian entrainment, long term potentiation, glutamatergic synapse, GnRH secretion and morphine addiction. Axon guidance and axonogenesis are important pre-requisites of cancer innervation and tumor exosomes containing EphrinB1 have been described to promote axonogenesis axonogenesis [48]. Tumor innervation is associated with poor clinical outcomes in several solid cancers including cervical and head and neck squamous cell carcinoma [48, 49]. Interestingly, our RT-PCR analysis showed presence of EPHB1 in the exosomal cargo of HPV-positive cervical cancer cells. Therefore, exosomal cargo of HPV-positive cells might be responsible for favouring neurite outgrowth. However, these assumptions require further functional validation.

Exosomes from HPV-positive cells contained high levels of TBC1D9, EVC2, RP11-11N9.4, NFIA, ANKS1B, LUZP1, MTRNR2L10 and MTRNR2L1 among the known coding transcripts. Although ectopic expression/export of these genes in cervical cancer or HPV infection remains unreported, some correlates suggest a potential tumor promoting role of some of them. EVC2 positively modulated Hedgehog (Hh) signaling pathway by forming complex with SMO protein and transduced Hh signaling in recipient breast cancer cells [50]. Notably, elevated Hh signaling is abberently and constitutively active in cervical cancer cells [32]. Similarly, enrichment of circANKS1B in HPV-positive cervical cancer exosomes may play a role in influencing cellular migration and invasion. circANKS1B increases the expression of transcription factor USF1, which upregulates TGF-β1 expression resulting in activated TGF-β1/Smad signaling and EMT in breast cancer [51]. Also, circANKS1B regulates FOXM1 expression and promotes cell migration and invasion by functioning as a sponge of the miR-149 in colorectal cancer [52]. ANKS1B was identified as a novel KRIT1 interacting protein that selectively controlled endothelial permeability [53]. Likewise, actin stabilising protein LUZP1, along with EPLIN, is known to alter cellular physiologies like EMT transition and cellular migration [54]. Therefore, differentially-exported exosomal transcripts from HPV-positive cervical cancer, may collectively contribute to tumor progression.

Cervical cancer being an HPV E6/E7-driven cancer, the HPV transcripts were highly expected in the exosomal compartment. Contrary to our hypothesis, a low enrichment of the viral transcripts was noted and the reads aligned sparsely and randomly over HPV16 and HPV18 reference genome. In contrast, abundant cellular expression of E6 and E7 was noted in both SiHa and HeLa cells. These transcripts were not detectable in SiHa exosomes, but HeLa exosomal RNA showed a specific splice variant HPV18 E6*I. Further, there was no alteration in exosomal E6/E7 transcript profile even if the cells were rendered to oxidative stress, a phenomenon known to induce exosome secretion and increase exosomal nucleic acid content [55, 56]. Nevertheless, detection of E6*I is a remarkable finding as it is the most dominant splice variant reported in primary HPV infection model [57] and increased significantly from 5-50% of all HPV mRNA in CIN2 to SCC samples during cervical cancer progression [58]. E6*I is known to play a prominent role in anti-apoptosis [59] and can potentially code for functional E7 protein [60]. Therefore, E6*I transcript which is predicted to have an important role in establishment of viral infection and early phase of viral propagation, may be executing its effect via exosome-mediated export and this observation demands further investigation.

## Conclusion

Overall, our study provides a detailed transcript profile of cervical cancer exosomes and discovered some remarkable differences in the pro-tumorigenic content and enrichment of mRNA of both cellular and viral origin. These signature mRNA along with other exosome-specific biomolecules can serve as disease biomarkers for cervical cancer progression. Our data showed a specific enrichment of truncated HPV18 E6*I mRNAs in HeLa exosomes, a lead which will serve as primer for detailed investigations addressing conditioning of constituent cells in the tumor microenvironment and will serve as a milestone in mRNA-based exosomal biomarker discovery in cervical cancer.

## Supplementary Information

**Additional file 1: Table SI.** List of antibodies used for immunoblotting (IB) experiments. **Table SII:** List of primers used in the study along with their sequence and amplicon size. **Table SIII.** Summary of raw sequence data and quality. **Table SIV.** Read alignment statistics with Combined Genome (GRCh38.p7, HPV16 & HPV18).

**Additional file 2: SF1.** Exosomal RNA characterization. **SF2.** Relationship between the assembled transcripts and closely related reference transcripts. **SF3.** KEGG Analysis of differentially expressed transcripts in exosomal RNA of HPV-negative vs. HPV-positive cervical cancer cells. **SF4.** Original uncropped blots for Fig. 1B

## Data Availability

The data sets used and or/analyzed during the current study are available from the corresponding author on reasonable request.

## Abbreviations

CaCx: Cervical cancer
HPV: Human papillomavirus
d-FBS: Depleted fetal bovine serum
BSA: Bovine serum albumin
PBS: Phosphate-buffered saline
BCA: Biocinchoninic acid
SDS: Sodium dodecyl sulphate
PVDF: Poly vinylidene fluoride
HRP: Horseradish peroxidase
ECL: Enhanced chemiluminescent
EDTA: Ethylenediaminetetraacetic acid
PAGE: Polyacrylamide gel electrophoresis
TEM: Transmission electron microscopy
NGS: Next generation sequencing
RIN: RNA integrity number
GO: Gene ontology
KEGG: Kyoto encyclopedia for gene and genomes
BLAST: Basic local alignment search tool
NCBI: National Center for Biotechnology
MF: Molecular Function
CC: Cellular Component
BP: Biological Process
PCA: Principal component analysis
CIN: Cervical intraepithelial neoplasia
RT-PCR: Reverse transcriptase PCR
lncRNA: Long non-coding RNA
TUG-1: Taurine upregulated-1
DNA: Deoxyribo nucleic acid
PCR: Polymerase chain reaction
RNA: Ribo nucleic acid
