# Supplementary material for: Transcriptome analysis of cervical cancer exosomes and detection of HPVE6*I transcripts in exosomal RNA
Source: BMC Cancer. 2022 Feb 11;22:164. doi: 10.1186/s12885-022-09262-4 (PMC8840784; doi:10.1186/s12885-022-09262-4)
Supplement: Supplementary file 1 — Additional file 1: Table SI. List of antibodies used for immunoblotting (IB) experiments. Table SII: List of primers used in the study along with their sequence and amplicon size. Table SIII. Summary of raw sequence data and quality. Table SIV. Read alignment statistics with Combined Genome (GRCh38.p7, HPV16 & HPV18). [file 12885_2022_9262_MOESM1_ESM.docx]

**Table SI: List of antibodies used for immunoblotting (IB) experiments.**

| **S. No.** | **Antibodies**  **(Mol. Wt.)** | **Source and Cat. No.** | **Origin and type** | **Dilution in IB** |
| --- | --- | --- | --- | --- |
|  | Alix (1A12)  (95 kDa) | Santa Cruz  (sc-53540) | Mu monoclonal IgG_1_ | 1:3000 |
|  | HSP 70  (70 kDa) | Abcam  (47455) | Mu monoclonal IgG_1_ | 1:3000 |
|  | Flotillin-2 (B-6)  (42 kDa) | Santa Cruz  (sc-28320) | Mu monoclonal IgG_1_ | 1:3000 |
|  | β-actin  (42 kDa) | Sigma  (A1978) | Mu monoclonal IgG_1_ | 1:5000 |
|  | Anti-mouse-HRP | Santa Cruz  (sc-2031) | Goat anti-mouse IgG | 1:5000 |
|  | Anti- Rabbit-HRP | Santa Cruz  (sc-2030) | Goat anti-rabbit IgG | 1:5000 |

**Table SII: List of primers used in the study along with their sequence and amplicon size.**

| **Primers** | **Sequence** | **Amplicon size** | **Annealing temp.** | **References** |
| --- | --- | --- | --- | --- |
| **R-HPV16E6** | F- 5’-AATGTTTCAGGACCCTACGG-3’  R- 5’-TCAGGACACAGTGGCTTTTG-3’ | 157 bp | 57.3 | [[1](#_ENREF_1)] |
| **R-HPV16E7** | F- 5’-TTTGCAACCAGAGACAACTGA-3’  R- 5’-GCCCATTAACAGGTCTTCCA-3’ | 214 bp | 57 | [[1](#_ENREF_1)] |
| **R-HPV18E6** | F-5’-GCGACCCTACAAGCTACCTG-3’  R- 5’-GTTGGAGTCGTTCCTGTCGT-3’ | 245 bp  428 bp | 62 | [[1](#_ENREF_1)] |
| **R-HPV18E7** | F-5’-GCATGGACCTAAGGCAACAT-3’  R-5’-TGTTGCTTACTGCTGGGATG-3’ | 322 bp | 58 | [[1](#_ENREF_1)] |
|  |  |  |  |  |
| **Beta-actin** | F-ATCCACGAAACTACCTTCAACTCCATC  R- CATACTCCTGCTTGCTGATCCACATC | 268 bp | 61 | [[2](#_ENREF_2)] |
| **EPHB1** | F- GTGTGCAATGTCTTCGAGCC  R- TCTGTGTAGATGCGATGGGC | 86 bp | 56 | [[3](#_ENREF_3)] |
| **EPHA6** | F- CATCCAGTTGGTCGGAATGC  R- AGTTTGGAAGAGGTCAGTTGTT | 202 bp | 58 | [[3](#_ENREF_3)] |

**References:**

1. Chang JT, Kuo TF, Chen YJ, Chiu CC, Lu YC, Li HF, Shen CR, Cheng AJ: **Highly potent and specific siRNAs against E6 or E7 genes of HPV16- or HPV18-infected cervical cancers**. *Cancer Gene Ther* 2010, **17**(12):827-836.

2. Raff T, van der Giet M, Endemann D, Wiederholt T, Paul M: **Design and testing of beta-actin primers for RT-PCR that do not co-amplify processed pseudogenes**. *BioTechniques* 1997, **23**(3):456-460.

3. Li S, Ma Y, Xie C, Wu Z, Kang Z, Fang Z, Su B, Guan M: **EphA6 promotes angiogenesis and prostate cancer metastasis and is associated with human prostate cancer progression**. *Oncotarget* 2015, **6**(26):22587-22597.

**Table SIII: Summary of raw sequence data and quality.**

| **Sample- ID** | **Number of Reads** | **Read Length** | **GC%** | **% Bases > Q20** | **% Bases > Q30** |
| --- | --- | --- | --- | --- | --- |
| SiHa-Exo | 41861174 | 151 | 44 | 98.80 | 84.97 |
| HeLa-Exo | 41604974 | 151 | 51 | 98.81 | 83.98 |
| C33a-Exo | 32186130 | 151 | 44.5 | 99.01 | 89.30 |

**Table SIV:** Read alignment statistics with Combined Genome (GRCh38.p7, HPV16 & HPV18)

| **Sample- ID** | **Reads after QC** | **Mapped reads** | **Mapped reads %** | **Uniquely mapped reads** | **Uniquely mapped reads %** | **Unmapped Reads** | **Unmapped Reads %** |
| --- | --- | --- | --- | --- | --- | --- | --- |
| SiHa-Exo | 37166256 | 36707542 | 98.77 | 32842616 | 88.37 | 458714 | 1.23 |
| HeLa-Exo | 35736678 | 33110372 | 92.65 | 20282718 | 56.76 | 2626306 | 7.35 |
| C33a-Exo | 29970450 | 29739990 | 99.23 | 26953750 | 89.93 | 230460 | 0.77 |
