# Supplementary material for: Transcriptome analysis of cervical cancer exosomes and detection of HPVE6*I transcripts in exosomal RNA
Source: BMC Cancer. 2022 Feb 11;22:164. doi: 10.1186/s12885-022-09262-4 (PMC8840784; doi:10.1186/s12885-022-09262-4)
Supplement: Supplementary file 2 — Additional file 2: SF1. Exosomal RNA characterization. SF2. Relationship between the assembled transcripts and closely related reference transcripts. SF3. KEGG Analysis of differentially expressed transcripts in exosomal RNA of HPV-negative vs. HPV-positive cervical cancer cells. SF4. Original uncropped blots for Fig. 1B [file 12885_2022_9262_MOESM2_ESM.pptx]

## Slide 1
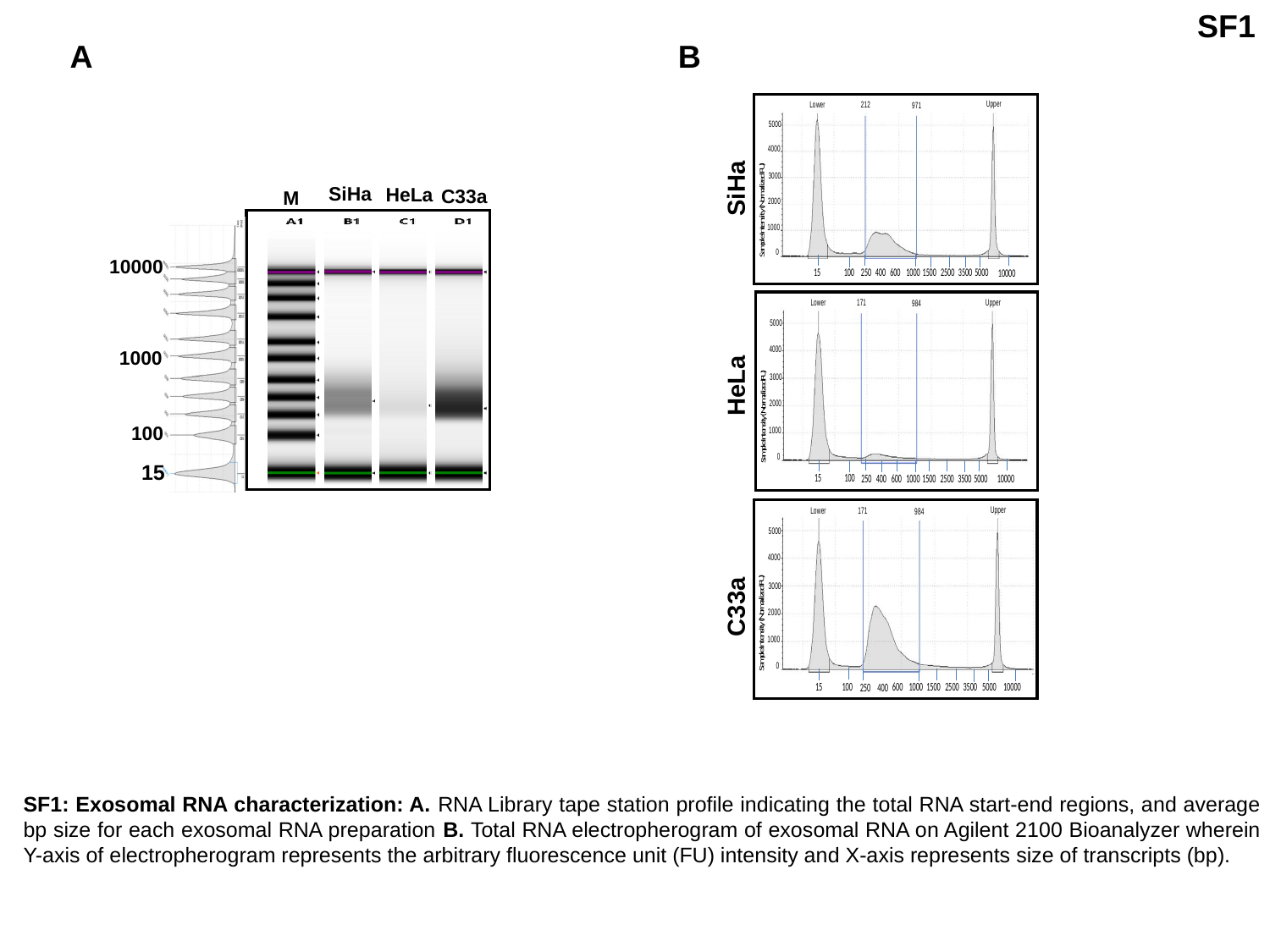

SF1
A
B
SiHa
SiHa
HeLa
C33a
M
10000
1000
HeLa
100
15
C33a
SF1: Exosomal RNA characterization: A. RNA Library tape station profile indicating the total RNA start-end regions, and average bp size for each exosomal RNA preparation B. Total RNA electropherogram of exosomal RNA on Agilent 2100 Bioanalyzer wherein Y-axis of electropherogram represents the arbitrary fluorescence unit (FU) intensity and X-axis represents size of transcripts (bp).

## Slide 2
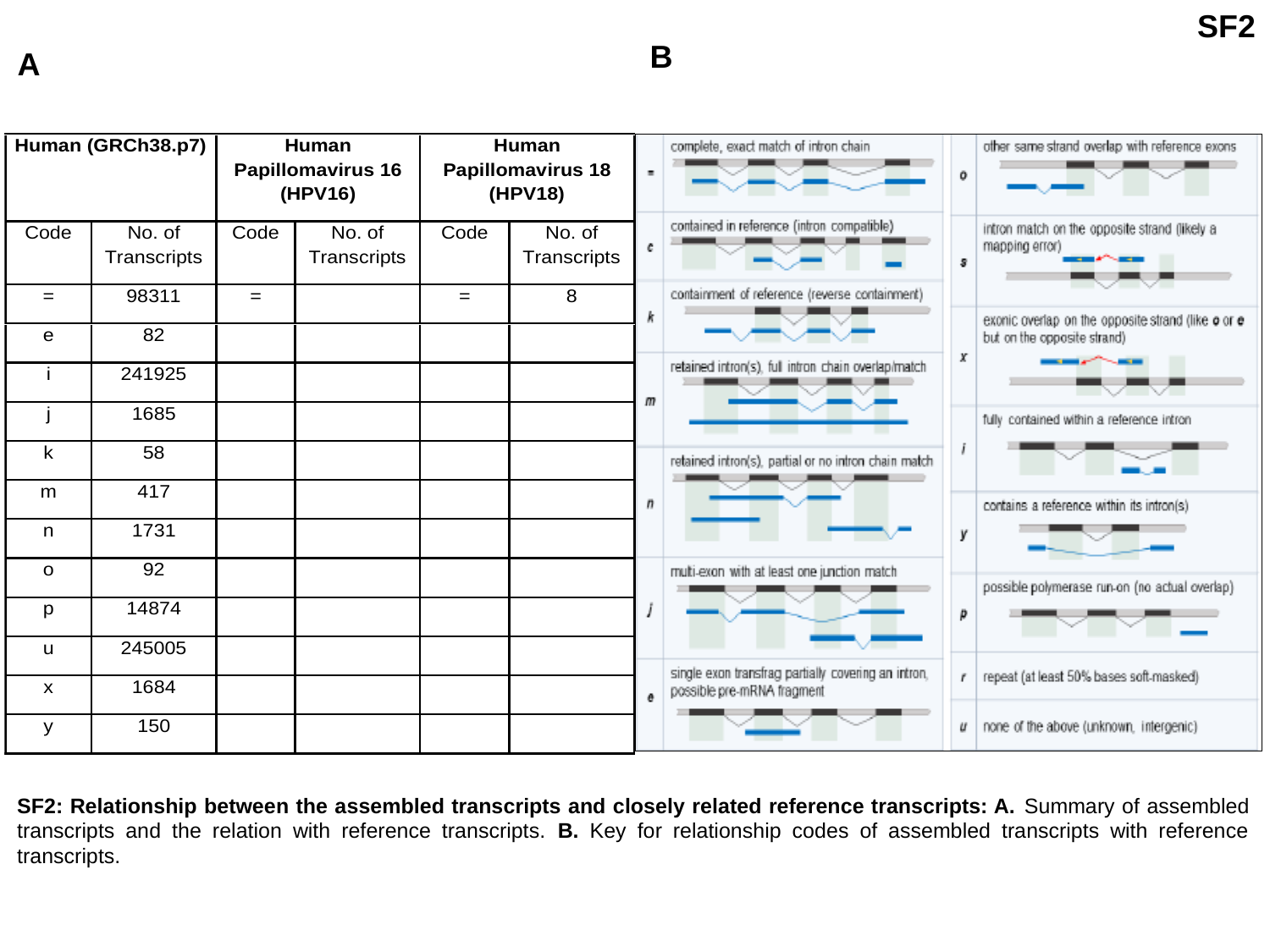

SF2
B
A
SF2: Relationship between the assembled transcripts and closely related reference transcripts: A. Summary of assembled transcripts and the relation with reference transcripts. B. Key for relationship codes of assembled transcripts with reference transcripts.

## Slide 3
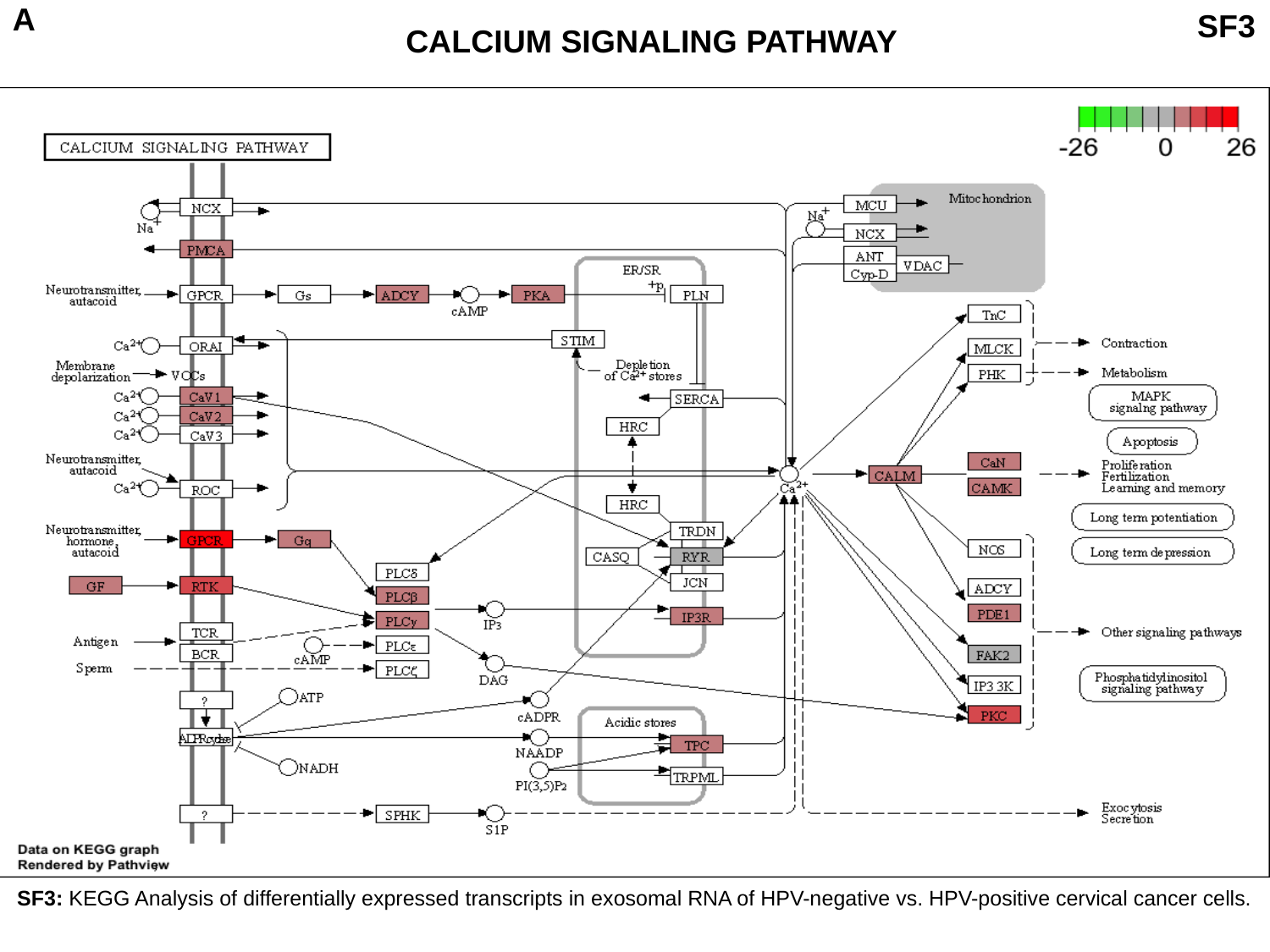

A
SF3
CALCIUM SIGNALING PATHWAY
SF3: KEGG Analysis of differentially expressed transcripts in exosomal RNA of HPV-negative vs. HPV-positive cervical cancer cells.

## Slide 4
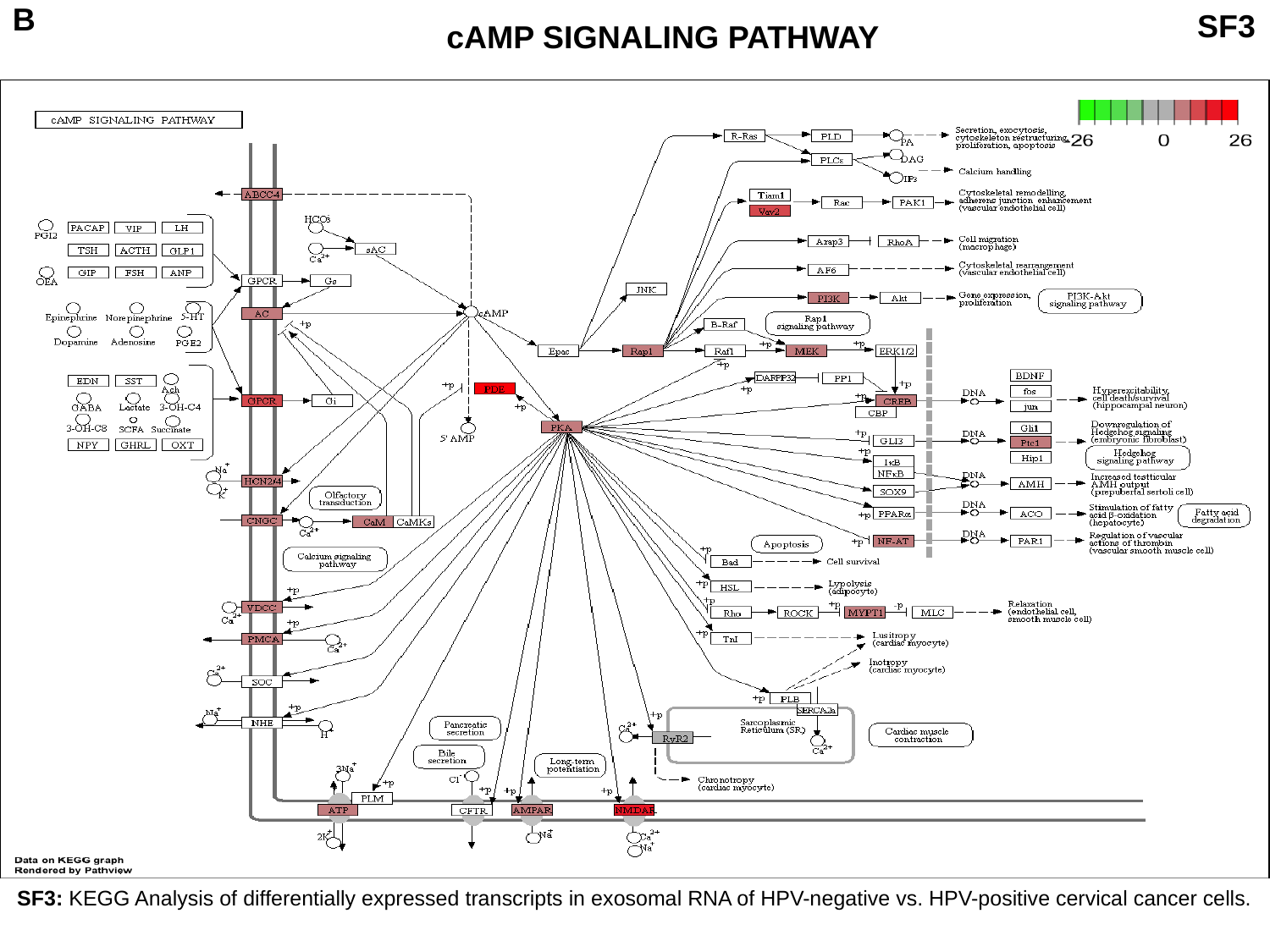

B
SF3
cAMP SIGNALING PATHWAY
SF3: KEGG Analysis of differentially expressed transcripts in exosomal RNA of HPV-negative vs. HPV-positive cervical cancer cells.

## Slide 5
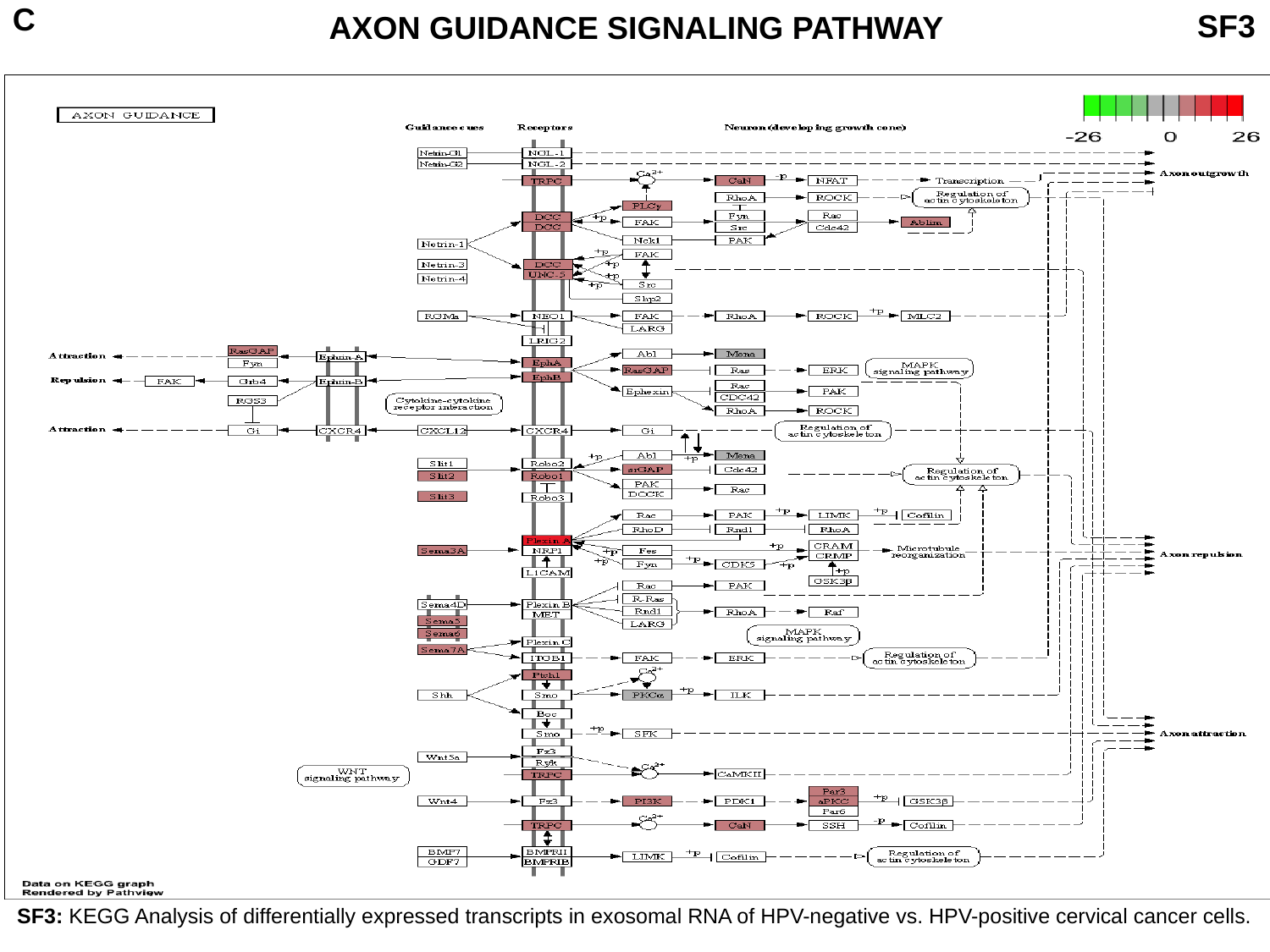

C
SF3
AXON GUIDANCE SIGNALING PATHWAY
SF3: KEGG Analysis of differentially expressed transcripts in exosomal RNA of HPV-negative vs. HPV-positive cervical cancer cells.

## Slide 6
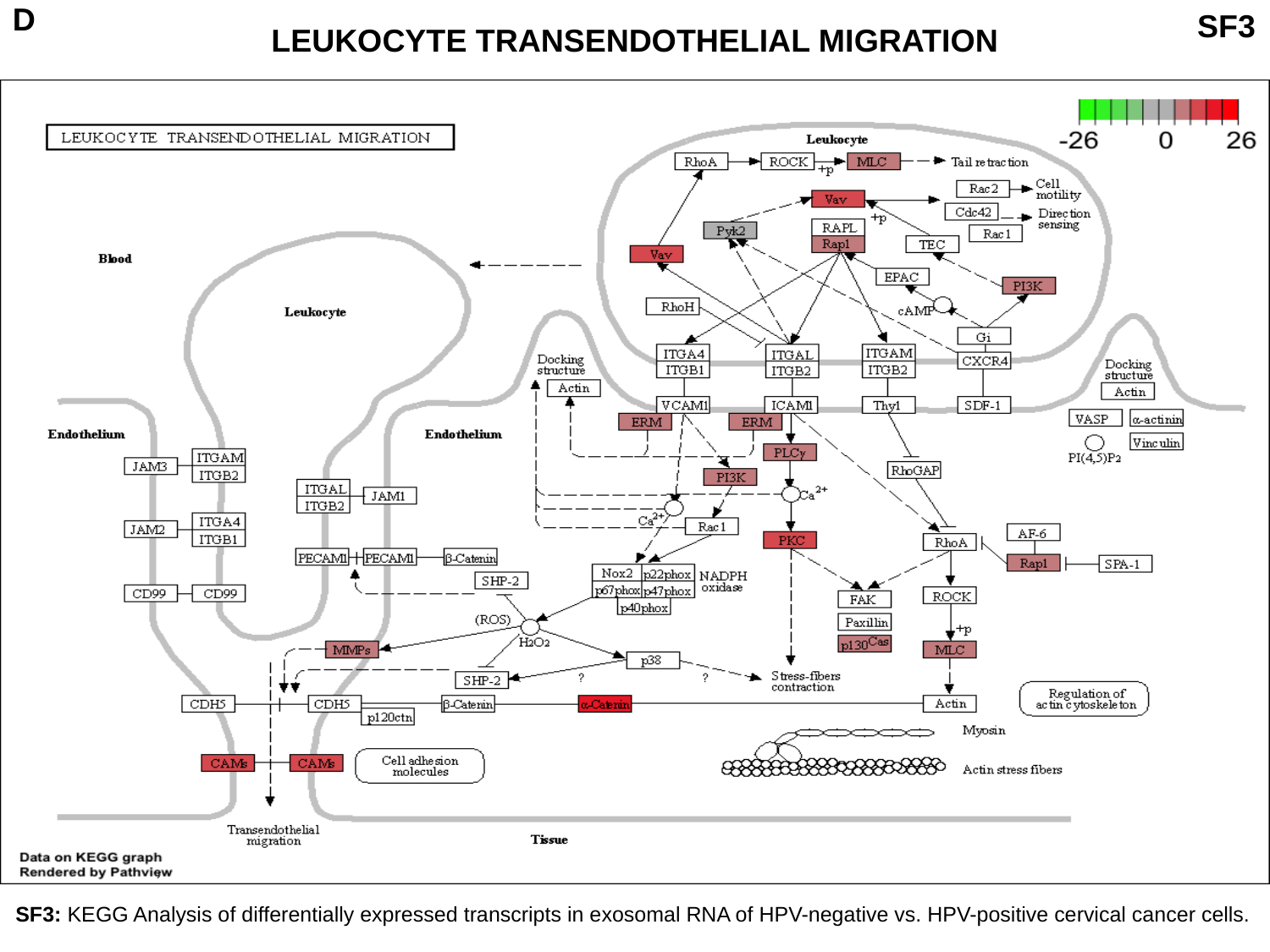

D
SF3
LEUKOCYTE TRANSENDOTHELIAL MIGRATION
SF3: KEGG Analysis of differentially expressed transcripts in exosomal RNA of HPV-negative vs. HPV-positive cervical cancer cells.

## Slide 7
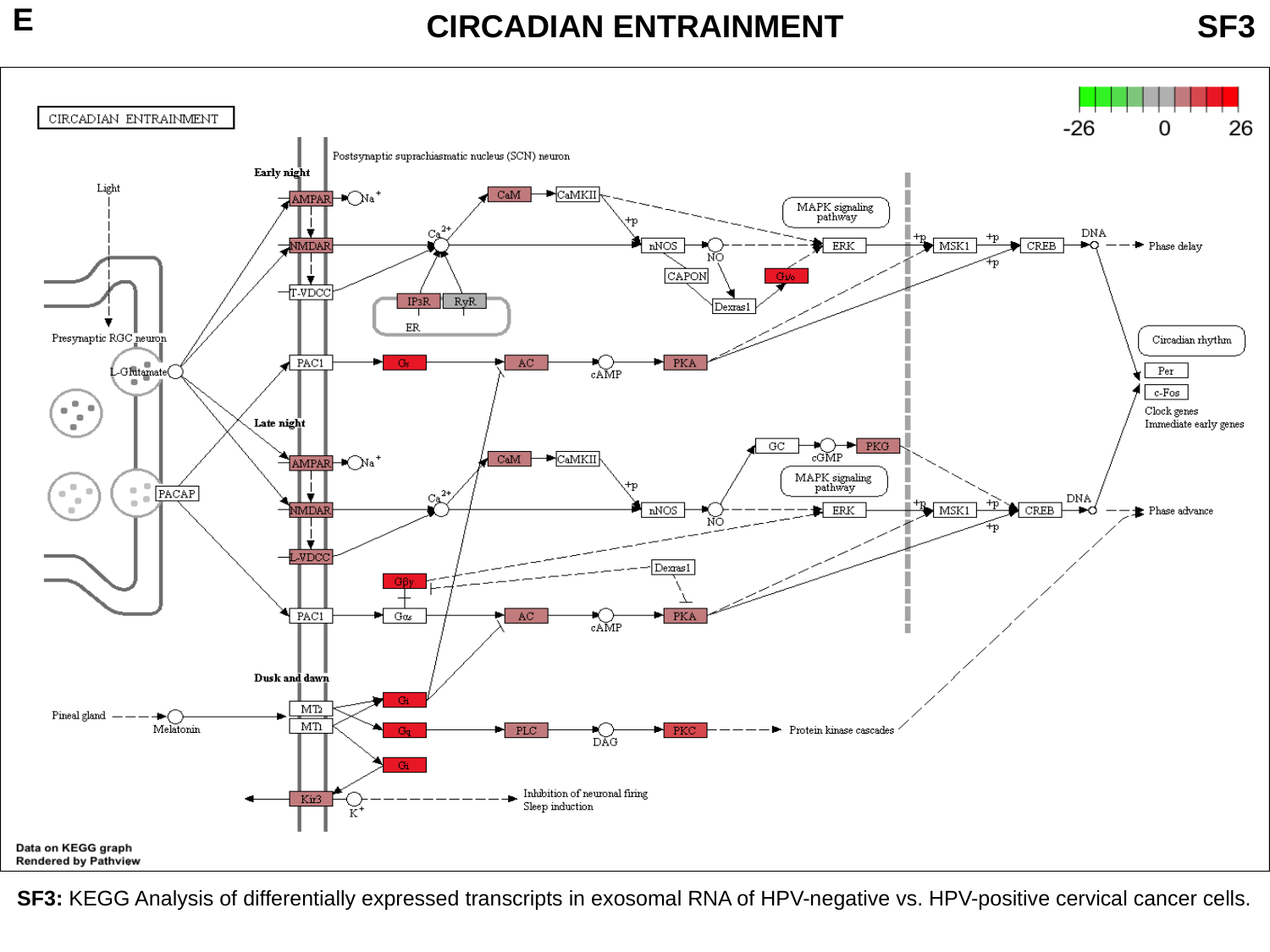

CIRCADIAN ENTRAINMENT
E
SF3
SF3: KEGG Analysis of differentially expressed transcripts in exosomal RNA of HPV-negative vs. HPV-positive cervical cancer cells.

## Slide 8
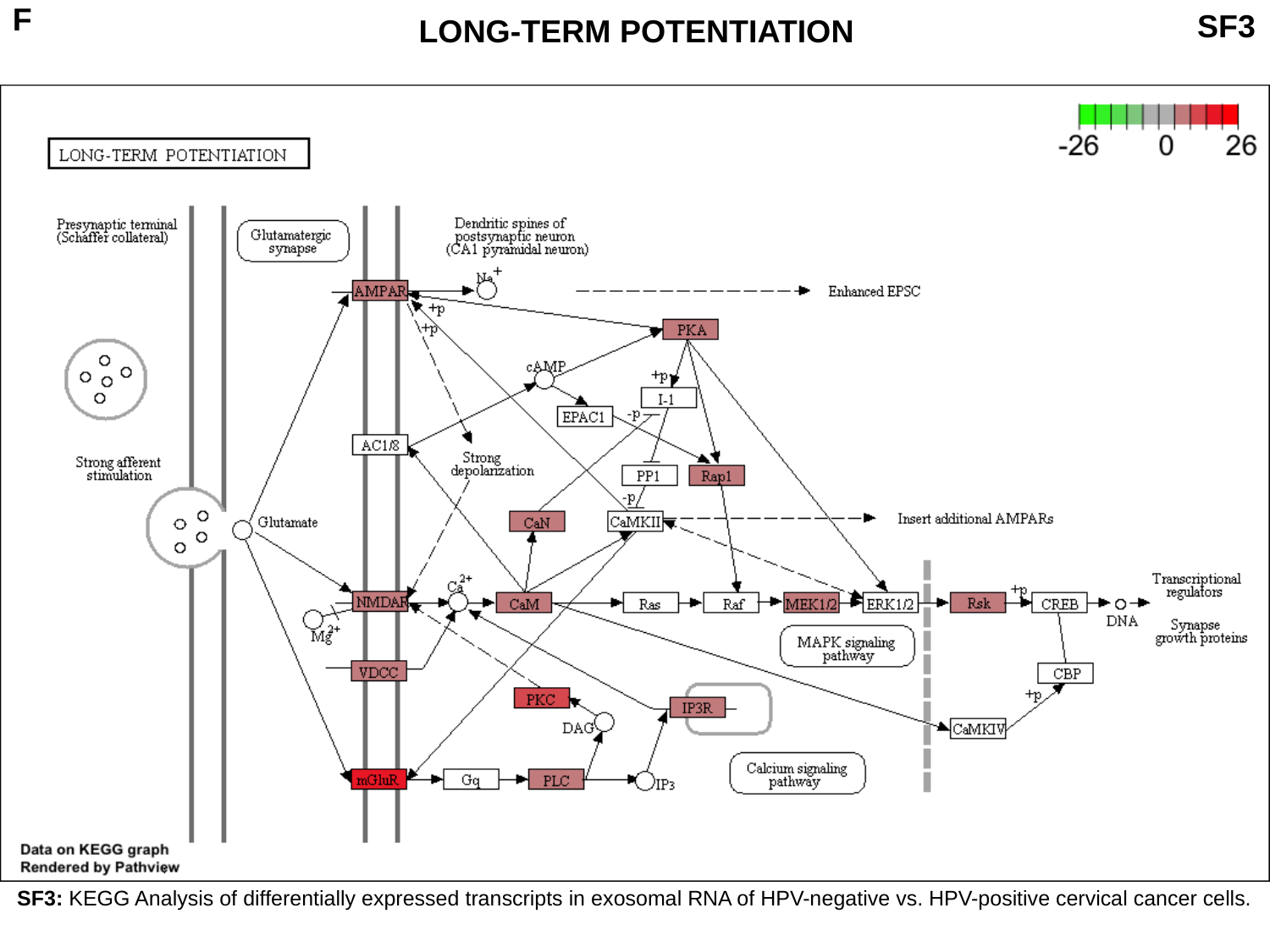

F
SF3
LONG-TERM POTENTIATION
SF3: KEGG Analysis of differentially expressed transcripts in exosomal RNA of HPV-negative vs. HPV-positive cervical cancer cells.

## Slide 9
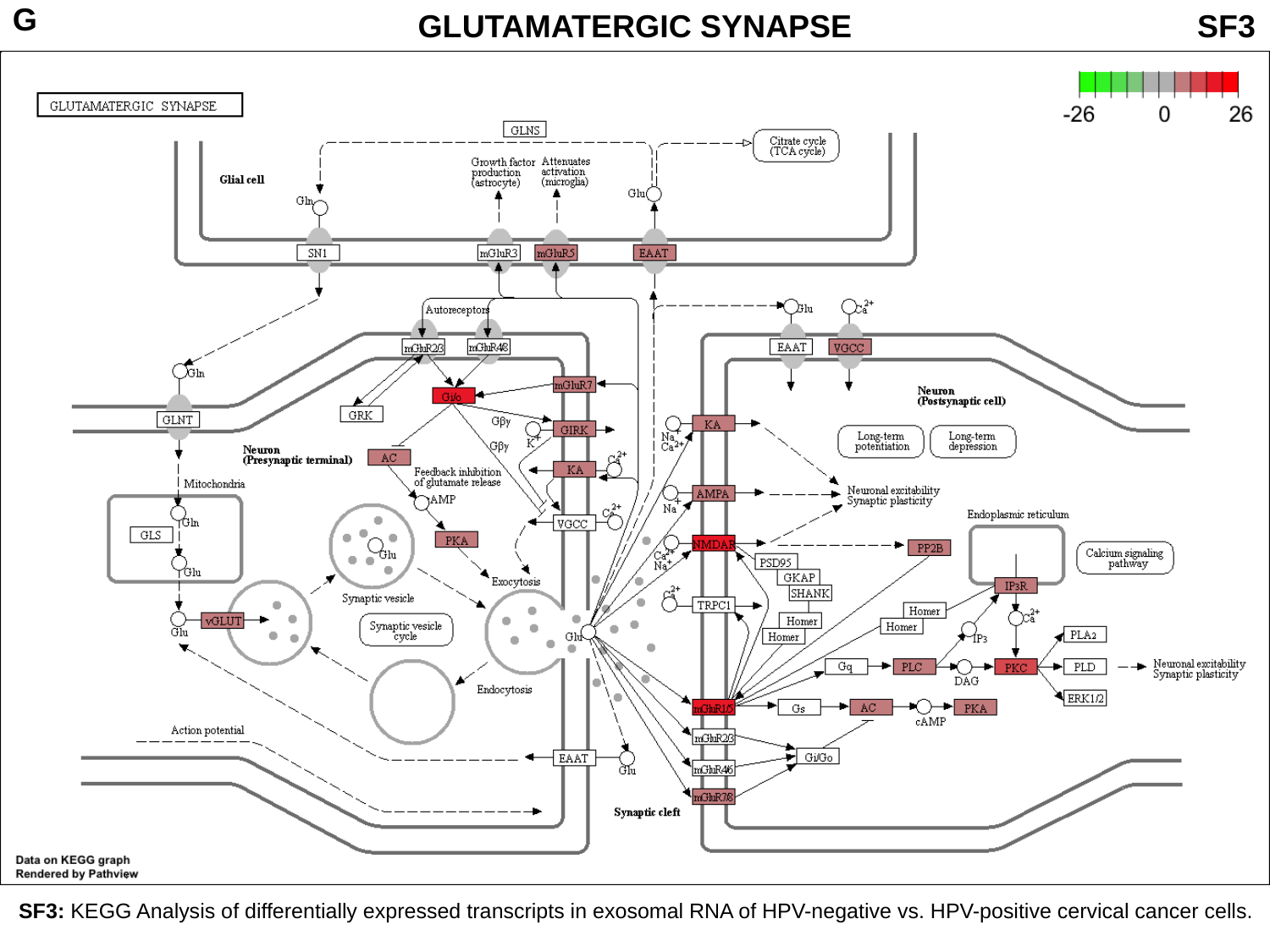

G
GLUTAMATERGIC SYNAPSE
SF3
SF3: KEGG Analysis of differentially expressed transcripts in exosomal RNA of HPV-negative vs. HPV-positive cervical cancer cells.

## Slide 10
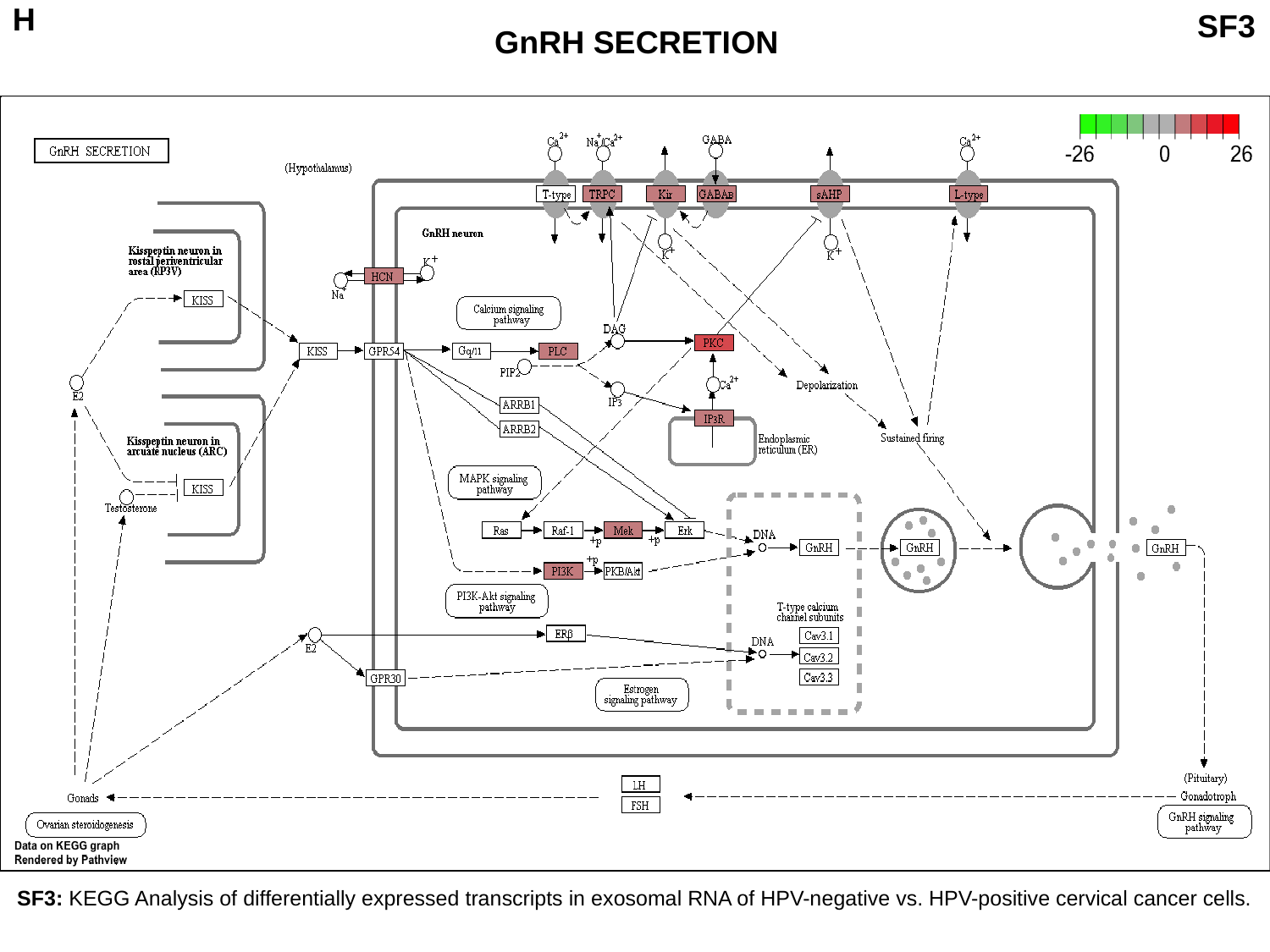

H
SF3
GnRH SECRETION
SF3: KEGG Analysis of differentially expressed transcripts in exosomal RNA of HPV-negative vs. HPV-positive cervical cancer cells.

## Slide 11
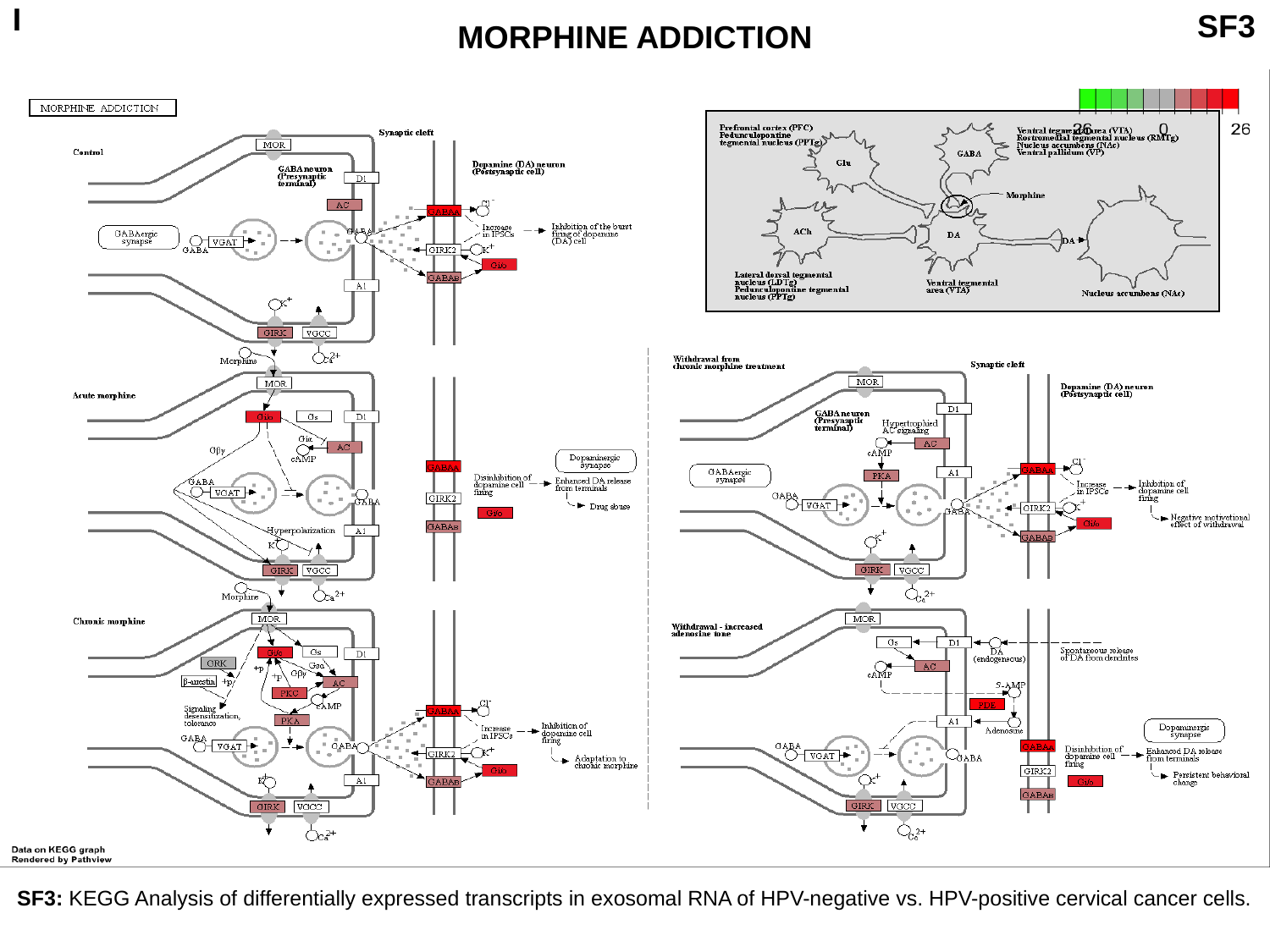

I
SF3
MORPHINE ADDICTION
SF3: KEGG Analysis of differentially expressed transcripts in exosomal RNA of HPV-negative vs. HPV-positive cervical cancer cells.

## Slide 12
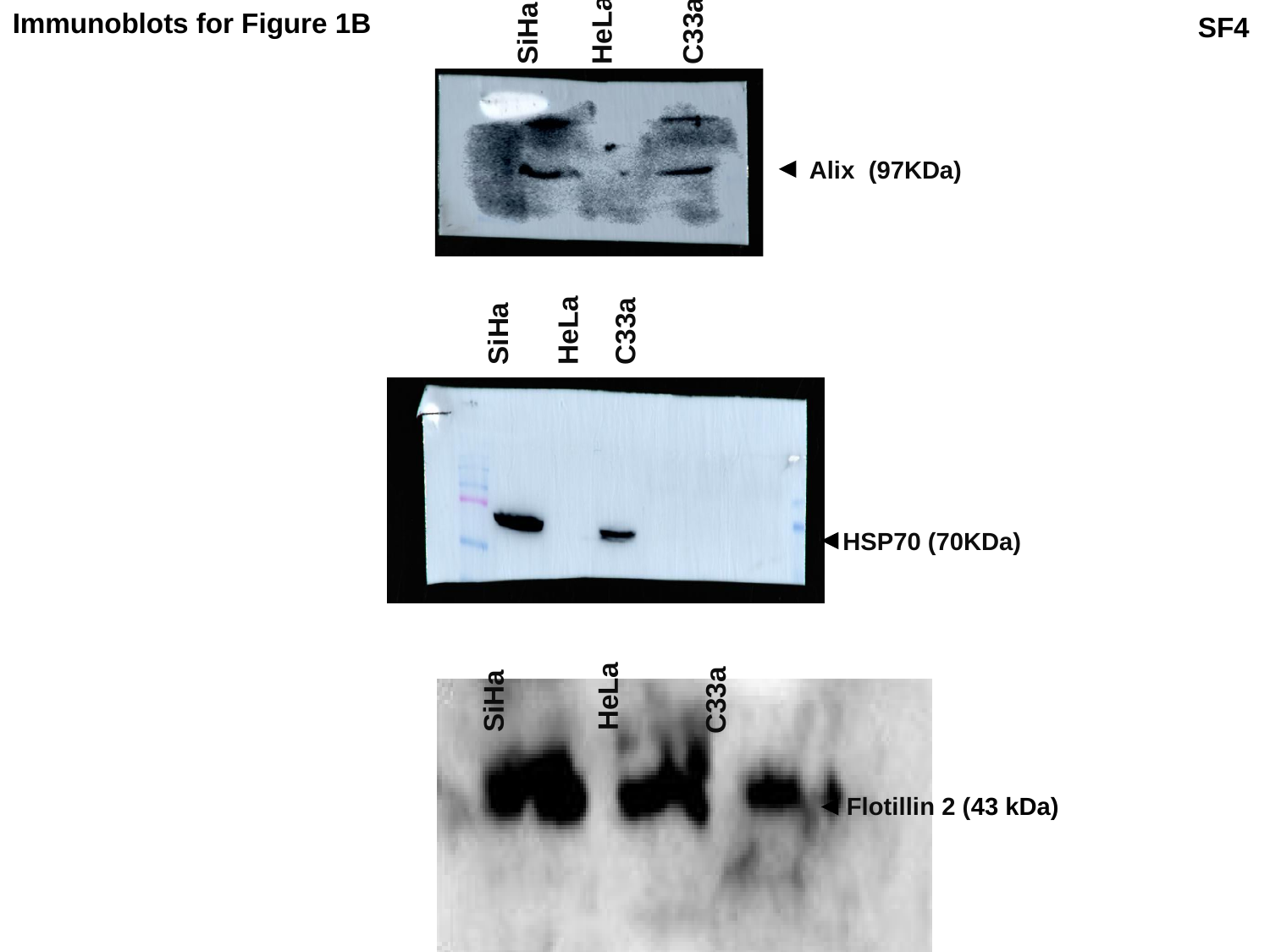

Immunoblots for Figure 1B
C33a
SF4
SiHa
HeLa
Alix (97KDa)
C33a
SiHa
HeLa
HSP70 (70KDa)
HeLa
C33a
SiHa
Flotillin 2 (43 kDa)
